# Unraveling the Impact of Extracellular Vesicle-Depleted Serum on Endothelial Cell Characteristics over Time

**DOI:** 10.3390/ijms25094761

**Published:** 2024-04-27

**Authors:** Luiz Fernando Cardoso Garcia, Pryscilla Fanini Wowk, Letusa Albrecht

**Affiliations:** 1 Laboratório de Pesquisa em Apicomplexa, ICC-Fiocruz-PR, Curitiba 81350-010, PR, Brazil; fgtc@outlook.com; 2 Laboratório de Virologia Molecular, Instituto Carlos Chagas, Fiocruz, Curitiba 81350-010, PR, Brazil; pryscilla.wowk@fiocruz.br

**Keywords:** extracellular vesicles, endothelial cells, fetal bovine serum, cell culture, proteomics

## Abstract

Extracellular vesicles (EVs) are produced by all kinds of cells, including endothelial cells. It has been observed that EVs present in fetal bovine serum (FBS), broadly used in cell culture, can be a confounding factor and lead to misinterpretation of results. To investigate this phenomenon, human brain microvascular endothelial cells (HBMECs) were cultured for 2 or 24 h in the presence of EV-depleted FBS (EVdS). Cell death, gene and protein expression, and the presence of EVs isolated from these cells were evaluated. The uptake of EVs, intercellular adhesion molecule 1 (ICAM-1) expression, and monocyte adhesion to endothelial cells exposed to EVs were also evaluated. Our results revealed higher apoptosis rates in cells cultured with EVdS for 2 and 24 h. There was an increase in *interleukin 8* (*IL8*) expression after 2 h and a decrease in *interleukin 6* (*IL6*) and *IL8* expression after 24 h of culture. Among the proteins identified in EVs isolated from cells cultured for 2 h (EV2h), several were related to ribosomes and carbon metabolism. EVs from cells cultured for 24 h (EV24h) presented a protein profile associated with cell adhesion and platelet activation. Additionally, HBMECs exhibited increased uptake of EV2h. Treatment of endothelial cells with EV2h resulted in greater ICAM-1 expression and greater adherence to monocytes than did treatment with EV24h. According to our data, HBMEC cultivated with EVdS produce EVs with different physical characteristics and protein levels that vary over time.

## 1. Introduction

The endothelium consists of a thin cell layer on the luminal surface of blood vessels. These cells play a crucial role in maintaining homeostasis and performing various activities at the cellular and biochemical levels, such as metabolizing vasoactive substances [1,2], synthesizing prostaglandins [3,4], presenting antigens [5,6], participating in blood clotting [7,8], and forming the blood–brain barrier [9,10]. Endothelial alterations can occur in response to various triggering agents, including bacteria [11,12], viruses [13,14], fungi [15], and protozoa [16], as well as noninfectious conditions, such as cardiac fibrosis [17], kidney diseases [18], or diabetes [19,20]. Researchers have highlighted the importance of cellular communication in response to various stressful stimuli, and the crucial participants in this process are extracellular vesicles (EVs) [21,22,23].

By convention, EVs comprise a diverse group of particles with a lipid bilayer that lack replicative capacity and are secreted by different cell types [24,25,26]. EVs play crucial roles in intercellular communication, serving as transporters of bioactive cargo between cells [27,28]. Once deemed mere cellular debris, these vesicles are now acknowledged as pivotal components in cell–cell communication [29,30,31,32]. EVs carry an array of factors from their cell of origin, including cytokines, chemokines, enzymes, nucleic acids, and surface molecules, influencing the behavior of recipient cells. They are instrumental in transmitting messages between cells, delivering proteins, nucleic acids (mRNAs, microRNAs), lipids, and metabolites [32,33,34].

EVs can be categorized based on various characteristics, with size and origin being the most common classifications [24,25]. Exosomes are smaller particles ranging from 40 nm to approximately 150 nm in diameter [35], while microvesicles typically measure between 100 and 500 nm [36]. To address the experimental challenges in distinguishing microvesicles and exosomes, the division of EVs into large (lEVs) and small (sEVs) vesicles has gained popularity [37,38,39,40].

EVs are present in various tissues, but those found in blood have been extensively studied due to their significant heterogeneity and potential role in mediating communication between distant cells [33,34,41]. Blood plasma EVs can be originated from platelets [42,43], red blood cells [44,45], leukocytes [46,47], or endothelial cells [21,48]. These particles induce various alterations in target cells based on their origin, concentration, or cargo [49]. EVs have been found to stimulate cell proliferation, angiogenesis, protein expression, molecular transport, and apoptosis [33,34,37,43,50,51]. EVs are also implicated in various pathological conditions like cancer, metabolic disorders, and inflammatory lung diseases, transmitting information between stromal, immune, and dysfunctional cells, thereby influencing pathogenesis and outcomes [52,53,54]. They are also associated with a wide range of other pathologies, including cardiovascular, renal, and musculoskeletal diseases [55,56,57]. The multifaceted roles of EVs in both well-known and emerging pathological conditions underscore their significance as key mediators in disease pathogenesis and potential therapeutic targets.

To study EVs, proper isolation of the particles through experiments is necessary [58]. However, it has been observed that EVs present in fetal bovine serum (FBS) used in cell culture can interfere with results and lead to erroneous information [59,60,61]. To avoid this interference, researchers use culture media supplemented with FBS depleted of EVs (EVdS) or culture media without FBS [59,60,62]. While both options improve the reliability of EV analysis, reducing important elements in FBS, including FBS-derived EVs, may directly impact cell growth and metabolism over time [60,63,64].

Serum-reduced or serum-free cell cultures refer to cells grown with partial or complete FBS deprivation. Chinese hamster ovary (CHO) cells and hybridomas [65,66] are common cells adapted to FBS-deprived cultivation where specific growth factors replace FBS, facilitating EV isolation without FBS contamination [59]. However, standardization for many other cell types has not been established, and their cell culture remains FBS dependent.

The isolation of EVs from cell culture supernatant can be performed using different culture timeframes for isolating EVs, ranging from 24 to 72 h, using medium containing EVdS [67,68]. Alternatively, some researchers prefer shorter culture times to obtain EVs, even though the yield may be lower, to prevent extended exposure of cells to a growth factor-deficient medium [69]. The choice of cultivation time depends on factors such as the selected model or stimulus. Therefore, selecting an appropriate methodology to obtain EVs is crucial for ensuring an accurate representation of the selected model. Studies of EVs from intracellular organisms are usually performed in short culture times to avoid obtaining EVs related to stress or apoptotic bodies [69,70,71]. This approach, coupled with studies showing that cultivation with EVdS can affect cell growth, raises questions about whether prolonged cultivation with EVdS produces EVs associated with cultivation-induced stress, compromising their suitability as models.

Hence, we present the first study comparing EV characteristics from human endothelial cells obtained at two different culture times, aiming to encourage other research groups to develop methodologies for the adequate isolation of EVs from other cell types.

## 2. Results

### 2.1. Endothelial Cells Grown in Media Depleted of EVs Exhibit an Increase in Early and Late Apoptosis

**Figure 1:**
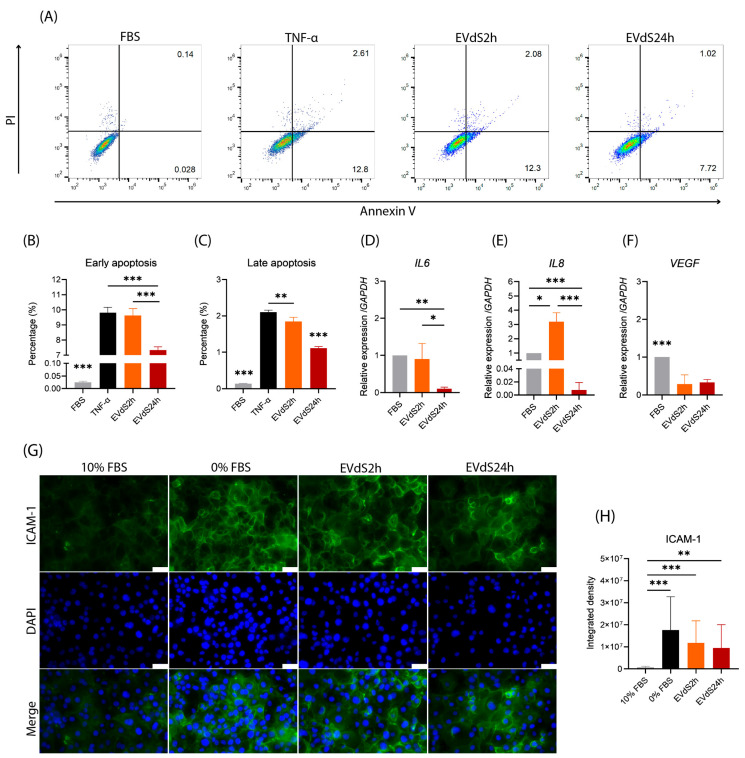
Activation of human brain microvascular endothelial cells (HBMECs) cultured in DMEM supplemented with extracellular vesicle-depleted fetal bovine serum (EVdS). HBMECs were cultured in 25 cm^2^ (T25) flasks with DMEM supplemented with 10% FBS or TNF-α (10 ng/mL). Afterward, the cells were suspended in sterile PBS, labeled with Annexin-V conjugated to phycoerythrin and propidium iodide (PI), and evaluated by flow cytometry (**A**–**C**). Annexin-V^+^/PI^−^ (early apoptotic cells) (**B**) and annexin-V^+^/PI^+^ (late apoptotic cells) (**C**) were graphically plotted. RNA from another cultured set of HBMEC was isolated for cDNA synthesis and quantification of *IL6*, *IL8,* and *VEGF* by qPCR (**D**–**F**). Intercellular adhesion molecule 1 (ICAM-1) expression was evaluated by staining HBMEC cultured in the absence of FBS (0%), 10% FBS, or EV-depleted FBS (EVdS) for 2 or 24 h. The cells were incubated with a primary anti-ICAM-1 antibody and a secondary anti-mouse conjugated to Alexa Fluor™ 488 (green), stained with DAPI (blue), and evaluated under a fluorescence microscope (**G**). The obtained images were evaluated using specific software, and the results are expressed as the integrated density (**H**). The data were obtained from three biological replicates and are expressed as the mean ± standard deviation (SD) (ANOVA/Tukey (**B**–**F**); Kruskal–Wallis/Dunn (**H**)); *: *p* < 0.05, **: *p* < 0.01; ***: *p* < 0.001. Scale bar, 50 μm (**G**).

We first assessed whether human brain macrovascular endothelial cells (HBMEC) cultured in Dulbecco Modified Eagle Medium (DMEM) supplemented with extracellular vesicle depleted serum (EVdS) exhibited evidence of endothelial activation or apoptosis. For this purpose, we cultured cells with EVdS for 2 (EVdS2h) or 24 h (EVdS24h) and evaluated apoptosis. As expected, few early Annexin-V-positive and IP-negative cells (annexin-V^+^/PI^−^) or late (annexin-V^+^/PI^+^) apoptotic cells were detected among the HBMEC grown on complete medium supplemented with whole FBS (Figure 1A,B). Before evaluating the treatments, we compared the apoptosis rate of HBMEC cultured in FBS for 2 or 24 h and did not observe any changes.

When the HBMECs were incubated in EVdS2h, the percentage of early apoptotic cells increased from 0.025% when the media was supplemented with complete FBS to 9.62% (*p*-value < 0.001). However, after 24 h, although the percentage of apoptotic cells remained greater than that of cells cultured in FBS (*p*-value < 0.001), this percentage decreased to 7.34% (*p*-value < 0.001) (Figure 1A,B). Tumor necrosis factor-alpha (TNF-α) is known for inducing endothelial cell apoptosis [72], and we used it as a positive control in this study. When comparing HBMECs cultured in EVdS2h medium with cells incubated with 10 ng/mL TNF-α, we observed a similar level of early apoptosis (*p*-value = 0.878). A similar phenomenon was observed for late apoptosis, where HBMECs cultured with EVdS2h exhibited an increase in apoptosis from 0.14%, observed in cells cultured with FBS, to 1.85% (*p*-value < 0.001). Among the HBMECs cultured with EVdS24h, 1.11% underwent late apoptosis, which was greater than that in the cells cultured with FBS (*p*-value < 0.0001) and lower than that in the cells cultured with EVdS2h (*p*-value < 0.0001) or TNF-α (*p*-value < 0.0001) (Figure 1A,C).

These investigations into the effects of DMEM supplemented with EVdS on HBMEC revealed significant evidence of endothelial activation and apoptosis. The experimental findings demonstrated a notable increase in both early and late apoptotic cells following exposure to EVdS, with distinct trends observed at 2 h and 24 h intervals. These observations suggest a time-dependent impact of EVdS on endothelial cell viability, with a more pronounced effect evident in the shorter duration. Furthermore, the observed differences between 2 h and 24 h exposure periods underscore the dynamic nature of the endothelial response to EVdS.

### 2.2. HBMEC Grown with EVdS Exhibit Increased Endothelial Activation

To evaluate the impact of EVdS over time on HBMEC, we quantified the expression of *Interleukin 6* (*IL6)*, *Interleukin 8 (IL8*), *suppressor of cytokine signaling 3* (*SOCS3*), *vascular endothelial growth factor* (*VEGF*)*,* and *OCCLUDIN* by quantitative PCR (qPCR). No significant differences were detected for *SOCS3* or *OCCLUDIN*. The absence of FBS-depleted EVs resulted in an approximately 10-fold reduction in *IL6* expression in cells cultured for 24 h compared to that in cells cultured with conventional FBS (*p*-value = 0.0098) (Figure 1D). A 3.19-fold increase in the expression of *IL8* was observed after 2 h of culture with EVdS (*p*-value = 0.0008), indicating a proinflammatory status. However, after 24 h, there was a decrease in *IL8* transcription to 0.0075 compared to that in HBEMCs in FBS (*p*-value = 0.0171) (Figure 1E). Compared with that in FBS-treated HBMEC, the expression of VEGF in HBMEC incubated with EVdS2h was 3.42-fold lower (*p*-value = 0.0025) and remained similar to that in HBMEC incubated with EVdS24h (*p*-value = 0.0034) (Figure 1F).

Alterations in the expression of intercellular adhesion molecule 1 (ICAM-1) were also evaluated. For comparison, as a control, we cultured HBMEC in the absence of FBS (0% FBS) (Figure 1G) [73]. We observed a change in the expression of ICAM-1 in cells cultured for 2 or 24 h with EVdS compared to that in HBMEC cultured for 24 h with FBS (*p*-value < 0.0001 for 2 h and *p*-value = 0.0039 for 24 h) (Figure 1H). This increase in expression reached a similar trend to that of HBMEC cultured for 24 h in DMEM without FBS (*p*-value > 0.05 for 2 and 24 h) (Figure 1H).

Our investigation into the effects of EVdS on HBMEC revealed distinct alterations in gene expression profiles over time. While no significant changes were noted for *SOCS3* or *OCCLUDIN*, the absence of FBS-derived EVs led to a marked transient proinflammatory state, evidenced by a significant increase in *IL8* expression after 2 h of exposure to EVdS, followed by a subsequent decrease after 24 h. Evaluation of ICAM-1 expression highlighted a notable increase following exposure to EVdS, suggesting a potential role in endothelial activation akin to cells cultured without FBS. These findings underscore the complex and time-dependent impact of EVdS on endothelial gene expression, implicating potential proinflammatory and angiogenic pathways.

### 2.3. EVs Isolated from HBMEC Cultured with EVdS for 2 or 24 h Exhibited Distinct Physical Characteristics and Protein Contents

**Figure 2:**
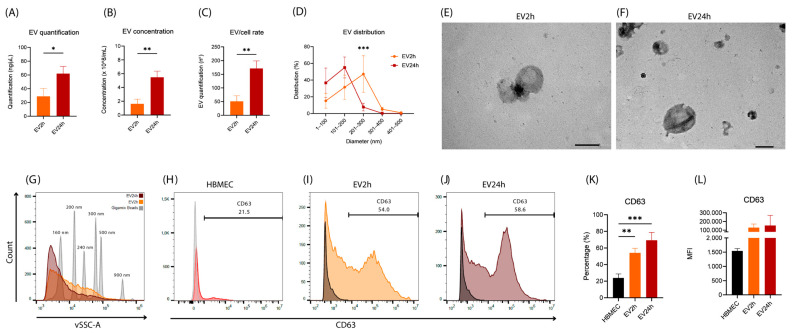
Extracellular vesicles isolated from cells cultured for 2 h (EV2h) and EV24h are different in size, concentration, and CD63 enrichment. EVs were obtained by differential centrifugation of the supernatant of HBMEC grown in DMEM supplemented with extracellular vesicle-depleted fetal bovine serum (EVdS) for 2 or 24 h. After isolation, indirect quantification of extracellular vesicles (EVs) was performed via fluorometry (Qubit^®^) (**A**). The size and distribution of the EVs and the particle/cell ratio were evaluated via nanoparticle tracking analysis (NTA) (**B**–**D**), and the morphology of the EV2h (**E**) and EV24h (**F**) populations was evaluated via transmission electron microscopy (TEM). Quantification of EVs and evaluation of CD63 enrichment were performed by flow cytometry. A histogram for the quantification of EVs was generated based on the signals detected by violet ray scattering (vSSC-A) (**G**). Evaluation of CD63 enrichment was performed with the detection of a primary anti-CD63 antibody and a secondary anti-rabbit IgG antibody (H + L) conjugated to Alexa Fluor^®^ 488 (**H**–**J**). The data obtained in (**H**–**J**) were plotted (**K**,**L**). The data were obtained from three biological replicates and are expressed as the mean ± standard deviation (simple *t*-test: (**A**–**C**); multiple (**D**) and ANOVA/Tukey (**K**,**L**)); *: *p*-value < 0.05, **: *p*-value < 0.01; ***: *p*-value < 0.001. Scale bar, 200 nm (**E**,**F**).

After observing that HBMECs exhibit different characteristics based on the period of incubation with EVdS, we hypothesized that there might be alterations in the EVs produced by these cells. According to the indirect quantification of EVs through protein fluorometrics, we detected a greater concentration of EVs (~62.0 ng/μL) produced in 24 h of culture (EV24h) than in 2 h (EV2h) (~29.1 ng/μL) (*p*-value = 0.02) (Figure 2A). The same difference was observed when EVs were directly quantified through nanoparticle tracking analysis (NTA) (Figure 2B) (*p*-value = 0.0038).

After evaluating the proportion of EVs per cell, we observed that after 2 h of culture, each cell secreted, on average, 51 EVs, while after 24 h, approximately 172 EVs were secreted (*p*-value = 0.0038) (Figure 2C). This value is approximate since many EVs were secreted and remained in the supernatant, while others were captured during the cultivation process. When evaluating the distribution of EVs, we found EVs with an average diameter between 100 and 400 nm for both culture conditions (Figure 2D). However, a greater concentration of EVs with a diameter between 200 and 300 nm was observed in the EVdS2h samples (*p*-value = 0.0017) (Figure 2D). When analyzing the EVs by transmission electron microscopy (TEM), we confirmed that the morphology of these particles and their size were consistent with the results obtained by NTA (Figure 2E,F).

We also used flow cytometry to evaluate the EVs. The distribution of EVs at both 2 and 24 h of cultivation resembled the results observed by NTA, with a concentration between 100 and 300 nm (Figure 2G). When analyzing the presence of Cluster of Differentiation 63 (CD63) (Figure 2H–J), a classic marker for EVs, we observed enrichment of this protein in the EV2h (~53.97%) and EV24h (~69.33%) compared to that in the HBMEC (~23.90%) (*p*-value = 0.0041 and 0.0005, respectively) (Figure 2K). Although the mean fluorescence intensity (MFI) of CD63 differed between EVs and HBMEC (~1543 for HBMEC, ~129,720 for EV2h, and ~153,812 for EV24h), the heterogeneity of the intensity obtained from EV24h was not significantly different according to analysis of variance (ANOVA) or Tukey’s test (*p*-value > 0.09) (Figure 2L).

**Figure 3:**
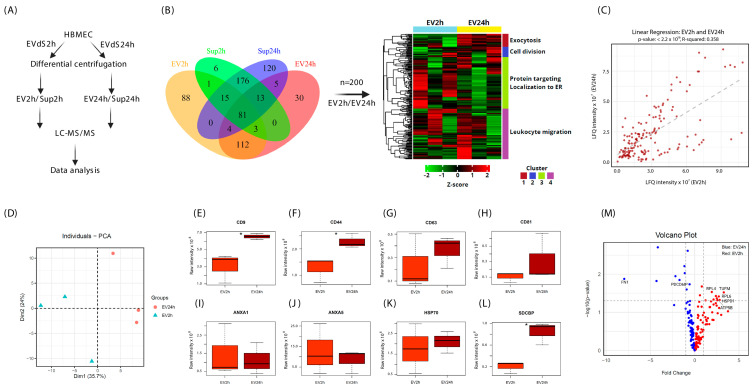
Extracellular vesicles isolated from cells cultured for 2 h (EV2h) and EV24h have different protein content. Extracellular vesicles (EVs) were obtained by ultracentrifugation from human brain microvascular endothelial cells (HBMECs) grown in DMEM supplemented with extracellular vesicle-depleted fetal bovine serum (EVdS) for 2 or 24 h. After isolation of the EVs, the proteins in the supernatant were precipitated with ammonium sulfate, and both the EV and the supernatant proteins were resolved on a polyacrylamide gel and analyzed using liquid chromatography–tandem mass spectrometry (LC–MS/MS) (**A**). The reference proteome for *Homo sapiens* was acquired from the UniProt database. The Boolean distribution of unique or common proteins between EVs and supernatants depleted from EVs at 2 h (Sup2h) or 24 h (Sup24h) was visualized in a Venn diagram, with 204 proteins common to both EV2h and EV24h. These proteins were normalized by the z-score and categorized on a heatmap (**B**). After evaluating the correlation between the EV2h and EV24h proteins using linear regression (**C**), the distance between replicates of EV2h and EV24h was determined by principal component analysis (PCA) constructed with 3 components (**D**). The raw intensities of CD9 (**E**), CD44 (**F**), CD63 (**G**), CD81 (**H**), ANXA1 (**I**), ANXA5 (**J**), SDCBP (**K**), and HSP70 (**L**) were graphically expressed in the EV2h and EV24h groups. The proteins that were differentially expressed at EV2h (**left**) and EV24h (**right**) are shown in a volcano plot (**M**). The data were obtained from three biological replicates. *: *p*-value < 0.05.

The proteomic analysis of the EVs followed the flowchart summarized in Figure 3A. Initially, the proteins present in the EV2h and EV24h were compared with the proteins contained in the EV-free supernatant. In the 2 h culture supernatant (Sup2h), 295 proteins were identified, 100 of which were associated with EV2h. In the 24 h culture supernatant (Sup24h), 414 proteins were identified. Of these, 103 were shared with EV24h, and 120 were exclusively observed in the supernatant. Additionally, 81 proteins were found to be common between EVs and supernatants, and 285 proteins were shared among the supernatants. When comparing EV proteins, we identified 304 proteins in the EV2h and 248 in the EV24h. Among those proteins, 200 were present under both conditions, 104 were observed only at EV2h, and 48 were present only at EV24h (Figure 3B). The proteins shared between EVs and supernatants (EV2h, Sup2h, EV24h, and Sup24h) are listed in Supplementary Table S1.

To analyze the differences between the two treatments, Z-score normalization was applied, and the results are presented as a heatmap of the four clusters. Using Euclidean distance, the samples were clustered, leading to the separation of samples based on their replicas. We observed greater expression of proteins related to protein signaling and localization to the endoplasmic reticulum (ER) in the EV2h group than in the control group, while proteins related to cell division and exocytosis were more highly expressed in the EV24h group (Figure 3B).

A linear regression model was used to explain 36% of the events (R squared = 0.358). The samples showed a strong positive correlation with a Pearson’s correlation coefficient of 0.598 and a *p*-value < 2.2 × 10^−16^ (Figure 3C). Principal component analysis (PCA) revealed consistency between replicates within each group and a difference between the two treatments (Figure 3D). Next, classic EV markers, including CD63, CD81, annexing-A1 (ANXA1), annexin-A5 (ANXA5), and heat shock protein-70 (HSP70), were evaluated (Figure 3G–J,L). No significant differences were observed between the treatments for these markers. However, the concentrations of CD9, CD44, and syndecan-binding protein (SDCBP) were greater in the EV24h group than in the EV2h group (*p*-values = 0.0259, 0.0466 and 0.0221, respectively) (Figure 3E,F,K). Proteins with constitutive expression, such as actin gamma-1 (ACTG1), glyceraldehyde-3-phosphate dehydrogenase (GAPDH), and tubulin A1 (TUBA1A), were also evaluated, but no significant differences between the treatments were detected (Supplementary Figure S2A–C).

Volcano plot analysis revealed that different mitochondrial and ribosomal proteins, such as mitochondrial Tu translation elongation factor (TUFM), ATP synthase F1 subunit beta (ATP5B), heat shock protein family D member 1 (HSPD1), ribosomal protein L4 (RPL4), and ribosomal protein L6 (RPL6), were differentially expressed in the EV2h, while apoptosis-related proteins, such as fibronectin-1 (FN1) and programmed cell death 6-interacting protein (PDCD6IP), were expressed in the EV24h (Figure 3M). Extended synaptotagmin-1 (ESYT1), prolyl 4-hydroxylase (P4HB), and protein disulfide isomerase A3 (PDIA3) were also differentially expressed in the EV2h. However, their names were omitted from the graph to facilitate visualization. Similarly, collagen type XVIII alpha 1 chain (COL18A1), milk fat globule-EGF factor 8 (MFGE8), transglutaminase 2 (TGM2), ADP-ribosylation factor 6 (ARF6), and clathrin heavy chain 1 (CLTC) were upregulated in the EV24h group.

**Figure 4:**
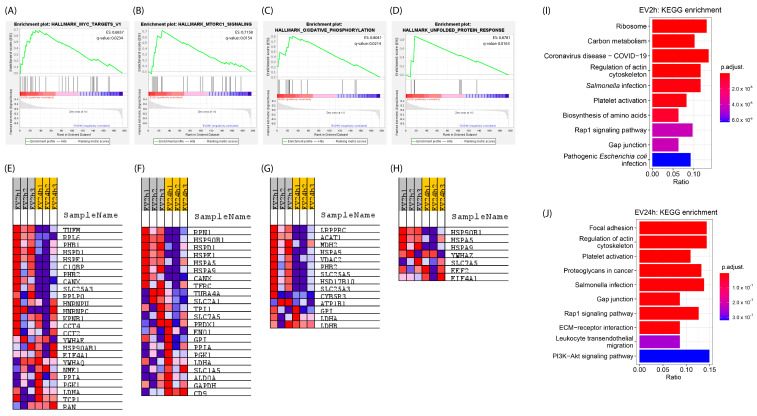
Extracellular vesicles isolated from cells cultured for 2 h (EV2h) and EV24h show different pathway enrichments. Extracellular vesicles (EVs) were obtained by ultracentrifugation from human brain microvascular endothelial cells (HBMECs) grown in DMEM supplemented with EV-depleted FBS (EVdS) for 2 or 24 h. The total protein content of the EVs was resolved on a polyacrylamide gel and analyzed using liquid chromatography–tandem mass spectrometry (LC–MS/MS). The reference proteome for *H. sapiens* was acquired from the UniProt database. Functional enrichment analysis (FEA) of all proteins identified at both EV2h or EV24h and pathways represented by a q-value less than 0.05 as MYC target (**A**), MTORC1 (**B**), oxidative phosphorylation (**C**), and unfolded protein response (**D**) were graphically plotted. The leading edge proteins are represented in a heatmap below their respective enrichment plots (**E**–**H**). The blue and red colors in the enrichment plot and heatmaps represent downregulated and upregulated proteins, respectively. Kyoto Encyclopedia of Genes and Genomes (KEGG) pathways of all proteins identified in the EV2h (**I**) or EV24h (**J**) that were functionally grouped by kappa score are shown in the bar graphs. The significance of each term was demonstrated with an adjusted *p*-value. The data were obtained from three biological replicates.

Using functional enrichment analysis (FEA), we observed the enrichment of specific pathways for proteins identified in the EV2h. We observed 25 proteins that were positively correlated (EC > 0) with the enrichment of MYC target genes (*q*-value = 0.0234) (Figure 4A,E), 23 related to the mechanistic target of rapamycin complex 1 (mTORC1) signaling pathway (*q*-value = 0.0154) (Figure 4B,F), 14 related to oxidative phosphorylation (*q*-value = 0.0214) (Figure 4C,G), and 7 related to the response to unfolded proteins (*q*-value = 0.0163) (Figure 4D,H). Proteins such as HSPs were shown to participate in multiple enrichments. No enrichment was observed for proteins identified in the EV24h (*p*-value and *q*-value > 0.05). The results of overrepresentation analysis (ORA) enrichment revealed 20 pathways with the highest enrichment for each sample group. Among the EV2h proteins, the pathways with the highest enrichment were related mainly to energy metabolism and ribosomes (Figure 4I). On the other hand, for the EV24h proteins, the highest enrichment was related to cell adhesion (focal adhesion and the Ras-related protein 1 (RAP1) signaling pathway), the cytoskeleton, and platelet activation (Figure 4J).

After normalization, we ranked the proteins identified in both the EV2h and EV24h groups and selected 50 proteins from each group according to the highest label-free quantification (LFQ) intensity indices. With this information, we constructed correlation matrices based on hierarchical clustering, and although many EV markers, such as CD9, annexin A2 (ANXA2), annexin A5 (ANXA5), (GAPDH), actin B (ACTB), and heatshock protein 90 alpha, class B, member 1 (HSP90AB1), were present in both matrices, the sets of correlations of the proteins of each EV population were distinct (Supplementary Figures S2B and S3A). After that, we selected the 50 proteins with the highest LFQ intensity indices in common between EV2h and EV24 and constructed a third correlation matrix in which, despite identifying all the aforementioned EV markers, they were correlated with more diffuse patterns than the previous matrices (Supplementary Figure S3C). We evaluated this pattern by developing a linear regression curve, but only 17% of the events were explained by the model, and the results were not statistically significant (R squared = 0.1663 and *p*-value = 0.07844) (Supplementary Figure S3D).

Our investigation into HBMEC’s response to varying incubation periods with EVdS revealed significant alterations in the characteristics of EVs produced by these cells. Both indirect and direct quantification methods showcased a notable increase in EV concentration after 24 h of culture compared to 2 h, alongside distinct patterns in the distribution and morphology of EVs and the maintenance of the expression of classical EV markers such as CD63. Proteomic analysis unraveled distinct protein profiles in EVs collected at different time points, highlighting significant pathways associated with protein signaling, cell division, and apoptosis. Moreover, FEA revealed specific pathways enriched in proteins identified in EV2h, indicating dynamic cellular responses. However, no enrichment was observed for proteins identified in EV24h, suggesting potential differences in cellular processes between the two time points. The differential expression of specific markers further underscores the complexity of HBMEC–EV interactions.

### 2.4. The Uptake of EV2h and EV24h by HBMEC Differs over Time

**Figure 5:**
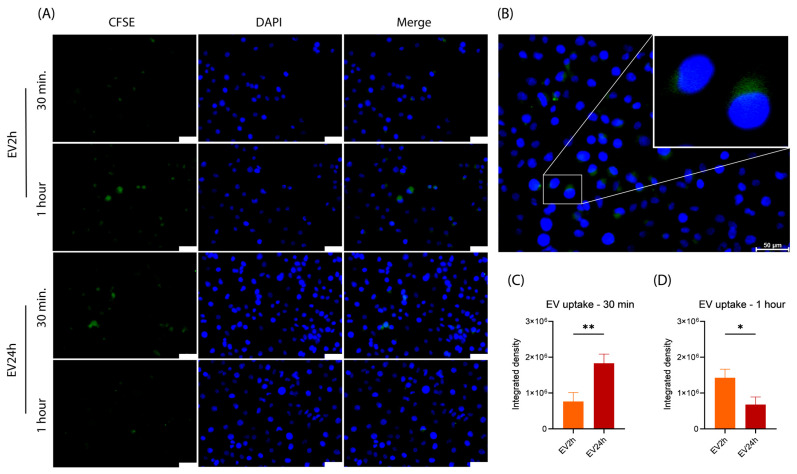
Extracellular vesicles isolated from cells cultured for 2 h (EV2h) and EV24h have distinct uptake ratios by HBMEC. Extracellular vesicles (EVs) were obtained from human brain microvascular endothelial cells (HBMECs) previously labeled with CFSE (green) and cultured in DMEM supplemented with EV-depleted FBS (EVdS). EVs were isolated through differential centrifugation. HBMECs were cultured in 24-well plates and incubated with EV2h or EV24h at a concentration of 100 ng/mL for 30 min or 1 h. Subsequently, the cells were washed, fixed with 4% paraformaldehyde, stained with DAPI (blue), and evaluated under a fluorescence microscope (**A**). Artificially enlarged image of EV2h uptake (**B**). The obtained images were analyzed using specific software, and the percentages of EVs taken up within 30 min (**C**) and 1 h (**D**) are expressed as the integrated density. The data were obtained from three biological replicates with five technical replicates and are presented as the mean ± standard deviation (*t*-test); *: *p* < 0.05, **: *p* < 0.01. The scale bar represents 50 μm (**A**,**B**).

Having verified that the number of HBMEC-derived EVs differed depending on the period of cultivation with EVdS, we aimed to evaluate alterations in the properties of these particles. Initially, we assessed the uptake rate of carboxyfluorescein succinimidyl ester (CFSE)-stained EVs. After comparing the uptake of EV2h with that of EV24h by HBMEC (Figure 5A), we observed a greater concentration of EV2h after 1 h of incubation and a greater concentration of EV24h after 30 min (Figure 5C,D). When evaluating the uptake of the particles, we observed their accumulation in specific regions of the cells instead of being diffusely distributed throughout the cytoplasm (Figure 5B). We also performed a comparison between the uptake of EV2h at 30 min and 1 h (*p*-value = 0.0342) and EV24h at 30 min and 1 h (*p*-value = 0.0205), and we observed opposite events, with an increase in EV2h uptake and a reduction in EV24h uptake over 1 h of incubation (Supplementary Figure S4). We also conducted the analysis after 4 h of incubation, but no fluorescence was detected in either experimental group. These findings highlight the dynamic nature of EV interactions and their potential significance in intercellular communication.

### 2.5. EV2h Enhances ICAM-1 Expression on HBMEC, Promoting THP-1 Adhesion

**Figure 6:**
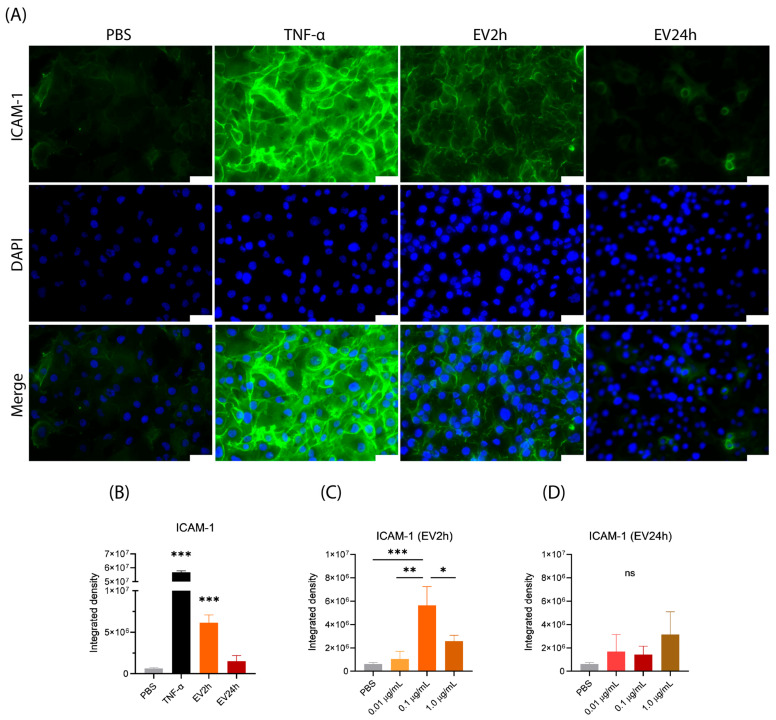
The expression of intercellular adhesion molecule 1 (ICAM-1) on human brain microvascular endothelial cells (HBMECs) is modulated by extracellular vesicles (EVs). EVs were obtained from HBMEC cultured in DMEM supplemented with EV-depleted FBS (EVdS) by differential centrifugation. HBMEC were grown in 24-well plates and incubated with EV2h, EV24h (100 ng/mL), TNF (10 ng/mL), or PBS for 24 h. A second experiment was conducted with cells incubated with extracellular vesicles isolated from cells cultured for 2 h (EV2h) or EV24h at concentrations of 0.01, 0.1, or 1.0 μg/mL. Following the incubation, both groups of cells were washed, fixed with 4% paraformaldehyde, and then incubated with a primary anti-ICAM-1 antibody. Subsequently, a secondary anti-mouse IgG antibody (H+L) conjugated to Alexa Fluor^®^ 488 was applied (green), and the cells were stained with DAPI (blue). The cells were then evaluated under a fluorescence microscope (**A**). The obtained images were analyzed using specific software, and comparisons between EV2h and EV24h (**B**) and between different concentrations of EV2h (**C**) or EV24h (**D**) are expressed as the integrated density. The data were obtained from three biological replicates with five technical replicates each and are presented as the mean ± standard deviation (ANOVA/Tukey). Statistical significance was determined as follows: *: *p*-value < 0.05; **: *p*-value < 0.01; and ***: *p*-value < 0.001. Scale bar, 50 μm (**A**).

Given that cells cultured with EVdS expressed more ICAM-1 on their surface, we hypothesized that EVs from HBMEC cultured under these conditions could also promote this alteration in the expression of adhesion molecules. We observed an increase in the expression of ICAM-1 in HBMEC incubated with EV2h compared to that in cells incubated with EV24h (*p*-value = 0.0003) or PBS (*p*-value < 0.0001) (Figure 6A,B).

We evaluated whether the modulation of ICAM-1 expression was related to the concentration of EVs applied during incubation in a dose-dependent manner. To test this possibility, we evaluated the expression of ICAM-1 in HBMEC stimulated with different concentrations of EV2h and EV24h. We observed that the HBMEC incubated with lower (0.01 μg/mL) or higher (1.0 μg/mL) concentrations of EV2h expressed less ICAM-1 than the cells incubated with 0.1 μg/mL EV2h (*p*-value = 0.0012 and 0.0142, respectively) (Figure 6C). The same effect was not observed in the HBMEC incubated with EV24h, in which even higher concentrations of EV24h (1.0 μg/mL) did not promote significant changes in ICAM-1 expression (*p*-value = 0.1424) (Figure 6D).

**Figure 7:**
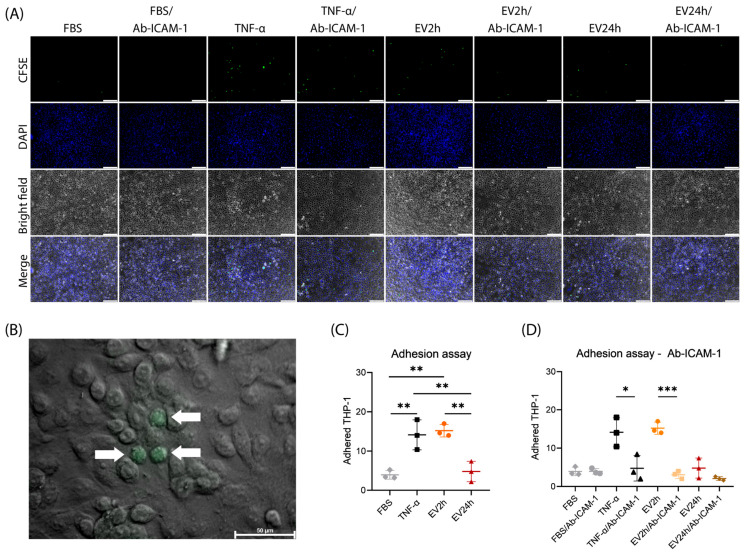
Adhesion of THP-1 cells to human brain microvascular endothelial cells (HBMECs) stimulated with extracellular vesicles (EVs) is modulated by intercellular adhesion molecule 1 (ICAM-1). EVs were obtained from HBMEC grown in RPMI supplemented with extracellular vesicle-depleted fetal bovine serum (EVdS) using a differential centrifugation method. HBMECs were grown in 24-well plates and incubated with EVs isolated from cells cultured for 2 h (EV2h) or EV24h (100 ng/mL), TNF (10 ng/mL), or PBS for 24 h (**A**). Another group of cells was subsequently incubated for 1 h with specific antibodies against ICAM-1 (Ab-ICAM-1) and washed with PBS to remove unbound antibodies. THP-1 cells maintained in RPMI were labeled with CFSE (green) and cocultured with HBMEC at a 1:1 ratio for 1 h. The cells were then sequentially washed, fixed with 4% paraformaldehyde, stained with DAPI (blue), and evaluated under a fluorescence microscope (**B**). The 400× magnified image shows THP-1 cells (arrows) adhered to HBMEC (**B**). The adherend THP-1 cells in the absence (**C**) or presence (**D**) of Ab-ICAM-1 were counted, and the results are expressed graphically. The data were obtained from three biological replicates and five technical replicates and are expressed as the mean ± standard deviation [ANOVA/Tukey (**C**) *t*-test (**D**)]; *: *p*-value < 0.05; **: *p*-value < 0.01; ***: *p*-value < 0.001. The scale bar represents values of 100 μm (**A**) and 50 μm (**B**).

We evaluated the adhesion of THP-1 cells to HBMEC that were incubated with FBS, TNF-α (10 ng/mL), EV2h, or EV24h (100 ng/mL) for 24 h (Figure 7A,B). Compared with those of cells cultivated in FBS, the adhesion of HBMEC incubated with EV2h (~14.13 cells) was greater than that of cells cultivated in FBS (3.933 cells) (~3.86-fold) (*p*-value = 0.0025) or EV24h (~3.17) (*p*-value = 0.0041) (Figure 7C). The increase in adhesion induced by EV2h was equivalent to that observed in cells incubated with TNF-α (*p*-value = 0.9511). On the other hand, compared with those in the FBS, the adhesion of THP-1 cells to HBMEC incubated with EV for 24 h did not significantly change (*p*-value = 0.9726) (Figure 7C).

When HBMECs were incubated with specific antibodies against ICAM-1 (Ab-ICAM-1), THP-1 adhesion was reduced in cells incubated with EV2h from approximately 14.13 cells to 4.733 cells (~4.96-fold) (*p*-value = 0.0004). No significant change in adhesion was observed in cells incubated with EV24h in the presence or absence of Ab-ICAM-1 antibodies (Figure 7D). We also evaluated the chemotactic effect of EV on THP-1 cells using a transwell experiment. We observed that, compared with Roswell Park Memorial Institute (RPMI) without FBS, EV2h had a stronger chemotactic effect (0% FBS) (*p*-value = 0.0236) (Supplementary Figure S5).

These findings show that EV2h increases ICAM-1 expression on HBMEC, promoting THP-1 adhesion. EV2h concentration affects ICAM-1 expression dose-dependently, with optimal effects at 0.1 μg/mL. EV2h significantly enhances THP-1 adhesion to HBMEC compared to EV24h or controls, akin to TNF-α. Blocking ICAM-1 antibodies mitigates EV2h-induced adhesion, confirming its role. These results highlight EV2h’s potential in modulating cellular adhesion, suggesting its relevance in inflammation and intercellular communication.

## 3. Discussion

Mostly, the isolation of EVs from different cells and tissues requires the cultivation of the target cells in culture medium supplemented with EV-depleted serum (EVdS) or even without this supplementation (0% FBS). FBS itself contains EVs that can interfere with subsequent analyses, justifying the need for EV-free medium [60]. Although this methodology is widely disseminated and used by different groups, it has been observed that cells cultivated in medium without FBS or with EVdS undergo changes in terms of the pattern of cell replication [63,74].

Aswad’s team observed that removing EVs from the culture medium affects the proliferation and differentiation of murine (C2C12), rat (L6), and human myoblasts. After including FBS-supplemented culture medium with EVs, both the morphology and transcription of different genes of these cells could be only partially recovered [63]. While Aswad’s team evaluated the impact of EVdS on muscle cells from various species, Eitan and colleagues investigated these effects on glioblastoma U87, HEK-293T, HeLa, human SY5Y, and mouse N2a neuroblastomas. After observing that FBS-EVs were internalized and associated with lysosomes in cells, the team discovered that when cultured with EVdS, all cell types, except U87, experienced a reduction in proliferation rate, with HeLa showing the most pronounced effect [74].

Eitan also contributed to another study examining the effects of EVdS on both Human Immunodeficiency Virus 1 (HIV-1) release and infectivity in human H9 cells. Interestingly, these effects were more pronounced after 2 days of culture. By the fourth day, virus proliferation rates approached those of cells cultured with complete FBS. When assessing the concentration of P24 antigen, the team observed higher levels in cells cultured with EVdS starting from the second day after infection. The team also conducted the experiment with PM1 cells and noted similar trends, albeit with a difference in rate emerging from the fourth day onward. Ultimately, they observed metabolic, surface marker, and gene expression changes in these cells [64].

Based on different evidence and suspicions about the effect of culturing cells with EVdS and its impact over time, we aimed to evaluate the culturing of endothelial cells with EVdS over 2 and 24 h. Our first studies were not related to EVs but to the whole supernatant. When evaluating the protein levels in the EV-free supernatant, we observed that when HBMECs were cultured in DMEM supplemented with EVdS for 2 h, the proteins were secreted and related to the cell cycle, replication, transcription, and energy metabolism (Supplementary Figure S6A). However, after they had been cultured for 24 h, they secreted a series of proteins associated with lysosomal acidification (Supplementary Figure S6B,C). Considering this and the potential negative influence of EVdS on cell culture [63,64], we investigated the evidence of cell stress upon cultivation with EVdS. Based on our primary results and Eitan’s works [64], we hypothesized that prolonged culture with EVdS could significantly affect cell development.

After evaluating cell death and transcriptional changes, we noticed that culture of HBMEC with EVdS for 2 h culminated in an increase in apoptosis to similar levels as that observed in cells incubated with TNF-α. We also observed an increase in the expression of *IL8* and a decrease in the expression of *VEGF*. Surprisingly, this dataset refuted our initial hypothesis that the cultivation of HBMEC with EVdS for 24 h would induce greater cellular stress. In situations of cellular stress, there is an increase in the expression of genes related to inflammatory interleukins, such as *IL6* and *IL8*, in the initial moments of the stimulus, followed by a reduction in their expression hours after the stimulus [75]. This phenomenon occurs, in many cases, due to an increase in the expression of genes associated with the regulation of the expression of these interleukins, for example, *SOCS3* [76]. However, we did not observe changes in *SOCS3* expression because, within 24 h of cultivation, the gene expression may have already been normalized.

The observation of fewer apoptosis events for 24 h may also be attributed to an event indicating the cells’ resilience to the new environment. Dela Paz and his team demonstrated that endothelial cells under shear stress exhibit a reduced rate of apoptosis, along with increased gene expression of *VEGF* modulated by Krüppel-like factor 2 (KLF2) [77]. Another significant study showcasing endothelial plasticity was conducted by Lacorre’s team. This study illustrated that within two days of culture, endothelial cells isolated from human tonsils rapidly lost their specialized characteristics, including the complete absence of venule-specific Duffy antigen receptor for chemokines (DARCs) and the HEV-specific fucosyltransferase Fuc-TVII [78]. In general, cellular plasticity is already a well-studied event observed in different cell types such as neurons, pancreatic, and salivary gland cells [79,80,81].

Different research groups have investigated the mechanisms underlying apoptosis in endothelial cells. Hogg and colleagues demonstrated that bovine vascular endothelial cells initiate apoptosis in response to various stimuli such as hypoxia, hyperoxia, hydrogen peroxide, the absence of FBS, and tissue growth factor B (TGF-B). It is noteworthy that the highest rates of apoptosis were observed in cells cultured without FBS [82]. Sheu’s team observed a similar phenomenon in human umbilical cord endothelial cells, where apoptosis could be triggered through a cascade starting with the activation of the phosphoinositide 3-kinase (PI3K) signaling pathway, ultimately leading to the activation of Caspase-3 [83].

Not only mechanisms inducing apoptosis in endothelial cells have been studied, but also regulation mechanisms. A seminal study conducted in 1999 demonstrated that endothelial cells cultured in a matrix composed of type 1 collagen exhibit upregulation of Bcl2, resulting in fewer apoptosis events compared to cells cultured in the absence of the matrix [84].

While Hogg utilized more severe stressors, such as complete deprivation of FBS, milder stimuli, such as the use of EVdS, could facilitate the process of cellular plasticity. To gain a better understanding of the events observed in our study, evaluating the expression rates of different genes related to apoptosis, such as PI3K, NFKB, CASP3, and B-cell lymphoma 2 (BCL2) signaling pathways, could provide crucial insights into cellular plasticity. Lastly, our qPCR analyses demonstrate the possibility that *VEGF* expression is not involved in this process.

Another molecule that may undergo changes in expression in response to endothelial activation is ICAM-1, an important molecule related to endothelial activation/dysfunction [85]. Its expression is closely associated with essential activities for maintaining life, such as inflammatory processes and the recruitment of leukocytes [73,86]. Endothelial cells exhibit a basal and constitutive expression rate of this protein. However, in the presence of various pathological events, such as infectious or inflammatory processes, an increase in the expression of this molecule may occur, which is associated with an activation/dysfunction process. Dysregulation in ICAM-1 expression can be triggered by different pathological events such as Dry Eye Disease [87], Crohn’s Disease [88], colorectal cancer [89], and hepatocellular carcinoma [90].

Studies conducted in 2000 demonstrated, using an ischemia/reperfusion model, that the expression of ICAM-1 in endothelial cells can be modulated by disrupting the genetic activation of Nuclear factor kappa B (NF-κB), thus potentially benefiting patients affected by ischemic shock [91]. Similarly, a study in 2013 also investigated the modulation of ICAM-1 expression in endothelial cells stimulated with TNF by inhibiting NF-κB activation. However, this group utilized polysaccharides as a potential agent to prevent endothelial injury associated with various diseases [92].

In our work, we observed an increase in ICAM-1 expression in the culture with EVdS, similar to that observed in cells cultivated in medium without FBS. Based on these findings, we believe that within 2 h of culture, HBMECs could undergo endothelial activation, and the isolation of EVs under these conditions could result in the collection of material that is less representative of a homeostasis model.

Given that endothelial activation can influence the characteristics of extracellular vesicles [93], we conducted a comprehensive evaluation of these particles. Following the guidelines set by the Minimal Information for Studies of Extracellular Vesicles (MISEV), we characterized the EVs in terms of size, concentration, morphology, and protein content [40,58]. Our studies revealed that after 24 h of cultivation, more EVs were obtained with a lower concentration of particles between 200 and 300 nm, indicating that the period of cell cultivation with EVdS not only affects the cells but also influences the vesicles produced by them.

CD63 is a classic marker of EVs for several cells [52,58]; thus, we assessed the enrichment of this protein in EVs. As other authors have reported alterations in the expression of CD63 between different cells in a physiological environment [52,94], we found that CD63 was enriched in both EV2h and EV24h cells. However, the percentage of EVs positive for CD63 varied depending on the duration of cell culture with EVdS. By understanding the heterogeneity of EVs produced by different cells [95] and the importance of CD63 as a modulating factor for cellular communication [96], we can hypothesize that when HBMEC are cultured for longer periods of time in EVdS, they will start to produce subpopulations of EVs that have more CD63 on their surface or start changing the protein pattern of different EVs. Thus, they could transmit a different message according to the cultivation time.

Stassen’s group conducted a study assessing the synthesis of CD63^+^/CD81^+^ EVs by various cells stimulated with cigarette smoke extract. They noted an increase in the expression of these two proteins in EVs derived from bronchial epithelial cells and lung fibroblasts but not from pulmonary microvasculature endothelial cells [97]. This disparity between our findings and those presented by Stassen suggests that endothelial cells can produce EVs with alterations in CD63 levels depending on the applied stimulus.

Proteomic analysis has proven valuable in evaluating metabolic pathway modulations as a whole or small variations in specific proteins in cells and EVs [98]. These analyses have played a critical role in multiomics experiments that use multiple assessments, enabling the detection of alterations that specific tests might not identify with the same precision [99]. Various factors can influence proteomic evaluations of EVs, such as conditions of cell cultivation [100] or EV isolation methodology [101]. As a result, different research groups often report diverse results. Several authors have identified close to 1500 proteins in EVs [102], while others might find fewer proteins—approximately 500 [103]. Nonetheless, the identification of classic EV markers, such as CD9, CD63, ANXA1, or HSP70, along with complementary techniques such as NTA, MET, or Western blotting, can enhance the reliability of the data [104,105,106].

Here, mass spectrometry-based evaluation of the EV protein content revealed significant differences between the culture conditions. While 204 proteins were present in both the EV2h and EV24h groups, most were differentially expressed. Additionally, we identified distinct expression patterns of other classic EV markers, such as CD9, CD44, and SDCBP. Specifically, EV2h exhibited numerous ribosomal proteins and proteins associated with carbon metabolism, whereas EV24h showed enrichment of pathways related to cell adhesion and platelet activation. A closer examination of the molecular interactions in the network revealed that EV2h contained all the enzymes involved in glycolysis (Supplementary Figure S7A). These findings suggest that HBMEC cultured for 2 h in EVdS undergo activation and share protein and energy machinery, enabling the survival of cells in a stressful environment.

While some authors have discussed the modulation of cellular energy metabolism by EVs, the exploration of this event often revolves around transcriptional alterations of genes such as NF-κB [107]. However, the presence of various ribosomal proteins in EVs from different cells has not been fully elucidated [108]. Although different authors have identified and described these proteins, little is known about their specific role and why they are present in EVs.

When conducting cell stimulation studies with EVs, it is essential to verify the uptake of these particles, the duration of their presence within target cells, and the appropriate EV concentration that does not overwhelm the cells and leads to less reliable data [27,95]. Direct labeling of EVs with a fluorophore and washing to remove residual reagents are classic methods for identifying EVs [109,110]. However, we observed an issue with the washing process when labeling EVs with Annexin-V (ANX5) followed by ultracentrifugation, as the residual dye precipitated together with the EVs (Supplementary Figure S8). Similar findings have been reported when PKH26 was used for EV labeling [111,112]. These observations indicate that the EV labeling process can generate particles of a certain size and fluorescence intensity, similar to those of EVs, leading to potential misinterpretations [113]. Although no studies have specifically evaluated this event with Annexin-V, our findings suggest that a similar phenomenon may occur with this fluorophore, other dyes, or even antibodies [114] used in EV labeling. Due to these complications resulting from the direct labeling of EVs, we opted for an alternative strategy. We prelabeled HBMEC with CFSE before initiating the EV collection process, allowing us to obtain EVs already marked with CFSE without any adjacent residual dye. However, we encountered two challenges with this technique: first, applying enough fluorophore to label many cells that will produce EVs; second, achieving weaker EV labeling than direct labeling. Despite these biases, we successfully detected the fluorescence emitted by the CFSE contained in the EVs. Our results revealed a lower fluorescence intensity in cells incubated with EV24h for 1 h than in those incubated for 30 min. Since CFSE is a dye intended for labeling dividing cells, we hypothesized that there was no reduction in the uptake rate after one hour of incubation with EV24h, but the CFSE contained in the EVs had already diffused through the cytoplasm of the recipient cell. This hypothesis is supported by the fact that we also failed to detect fluorescence in cells incubated for 4 h with either EV2h or EV24h.

Other research groups, such as Bonsergent and collaborators, have shown that the uptake of EVs by HeLa cells begins within approximately 30 min and continues for up to 20 h. In a dose-dependent study, the group also assessed the uptake rate of EVs at concentrations similar to those in our study (100 ng/mL), revealing lower uptake rates and a decreased concentration of EVs inside the cells [27]. Toribio’s group devised strategies to examine the uptake of EVs by SUM159 cells. In their investigation, Toribio observed that EV uptake commenced around 30 min, peaked at 2 h, and remained stable until the end of the 4-h experiment [115].

Although these studies share some similarities, they employed high concentrations of EVs (ranging from 1–10 μg/mL and 40–80 μg/mL), potentially leading to continuous capture by cells until exhaustion rather than sustained presence within the cytosol for hours. To address this, we utilized lower EV concentrations to prevent saturation of the system. An optimization strategy for this study would involve removing the supernatant containing EVs after 20–30 min to assess the uptake of EVs captured solely during this period. Another strategy could be live-cell imaging [116].

After observing the difference in EV uptake over the course of cell culture with EVdS, we suspected that the particles would exhibit other differences in their biological activities. To investigate this phenomenon, we assessed the alteration in ICAM-1 expression in HBMEC stimulated with EV2h or EV24h. We found that HBMEC cultured for 2 h with EVdS not only exhibited an increase in ICAM-1 expression but also that their EVs were able to modulate ICAM-1 expression in other cells. However, this effect was observed only for EV2h at specific concentrations. Neither lower nor higher concentrations of EV24h modulated ICAM-1 expression. Previous studies have described the dose-dependent effects of EVs on the permeability, proliferation, and migration of human umbilical cord endothelial cells (HUVECs) [117,118]. However, unlike our results, those authors did not observe a reduction in these events with higher concentrations of EVs. We speculated that events other than those we studied were being observed, and additionally, the authors might have used higher concentrations of EVs than the concentrations used in our study (15, 30, and 60 μg/mL and 10, 40, and 80 μg/mL). Another research group conducted similar experiments and observed an EV-mediated increase in HUVEC ICAM-1 expression [119]. They reported that high concentrations of different fractions of EVs promoted increased expression of ICAM-1. However, the authors also used concentrations as high as 1.0 × 10^8^ particles/mL, equivalent to approximately 3800 ng.

Unlike authors who used higher concentrations of EVs, we deliberately chose to use lower concentrations of EVs for two reasons: first, by determining the average number of EVs produced per cell, we could accurately assess the concentration of EVs/mL in the environment and use a concentration that more reliably mimics the biological model under study. Second, the uptake assay demonstrated that using 100 ng/mL EVs did not lead to the saturation of cells with excessive particles. Although these differences could be observed between studies, the concentration of EVs used will depend on the experimental design.

The protein content of EV2h may reflect a state of endothelial activation, whereas the protein content of EV24h, related to cell junctions and interacting proteins, does not necessarily indicate a cumulative effect of the stress caused by EVdS. Instead, these findings suggest possible cellular resilience to new environments deprived of nutrients. It was shown that murine endothelial EVs could modulate endothelial activation [120]. The authors observed that curcumin-loaded EVs altered the expression of ZO-1 and VE-cadherin in endothelial cells not only in response to EVs associated with curcumin but also in combination with EVs without the compound. However, in the mentioned work, EVs were isolated after 72 h of culture with EVdS, which might be a period during which endothelial activation caused by the switch from FBS to EV-depleted medium was already controlled by the cells.

In endothelial cells, activation by different stimuli can lead to the expression of adhesion molecules on the cell surface, like ICAM-1 [91]. This leads to an increased rate of margination, rolling, and diapedesis of neutrophils or monocytes to the site of infection or inflammation in an organism. In vitro models can mimic this event by incubating endothelial cells with the particle or microorganism of interest, followed by incubation with leukocytes such as neutrophils or monocytes [121,122].

Studies have shown that EVs from a proinflammatory environment can modulate ICAM-1 expression in endothelial cells and increase the adhesion of neutrophils [86] or monocytes [73] to these cells. Although these studies used HUVECs, the findings are likely applicable to HBMEC since both are endothelial cells. Hosseinkhany’s group [73] reported that the increase in adhesion was mediated by ICAM-1, which aligns with our observations in HBMEC. These findings support the hypothesis that endothelial cells can influence the gene and protein expression of other endothelial cells via EVs, even at a distance from the site of the stimulus. To understand the differences in ICAM-1 expression mediated by EVs, we performed ORA and found that both EV2h and EV24h contained proteins related to leukocyte adhesion, such as ICAM-1, ACTIN, Ezrin, Radixin, and Moesin (ERM) and Integrin Beta 1 (ITGB1, also called CD29), at similar concentrations (Supplementary Figure S7B,C). Thus, we do not believe that the increase in cell adhesion is solely due to the transfer of these proteins via EVs. Instead, we considered three possible explanations for this difference. First, the increase in ICAM-1 expression could be due to the TNF receptor (TNFr)-mediated signaling cascade. TNFr is a promiscuous receptor capable of initiating cellular stress processes mediated by various particles [123]. This hypothesis is supported by the observed increase in ICAM-1 expression and adhesion in TNF-α-stimulated cells. Second, miRNAs such as miR-221 can be transferred via EVs [124]. miR-221, which is transferred via EVs, can bind to the 3′UTR and suppress the translation of *ICAM1*, resulting in reduced protein expression [125,126]. Finally, the transfer of several proteins related to the transcription and translation machinery via EVs could favor an increase in the expression of different proteins related to a stressful environment. This hypothesis is supported by the identification of several ribosomal subunits, glycolysis enzymes, initiators, and transcription factors in EV2h.

## 4. Materials and Methods

**Figure 8:**
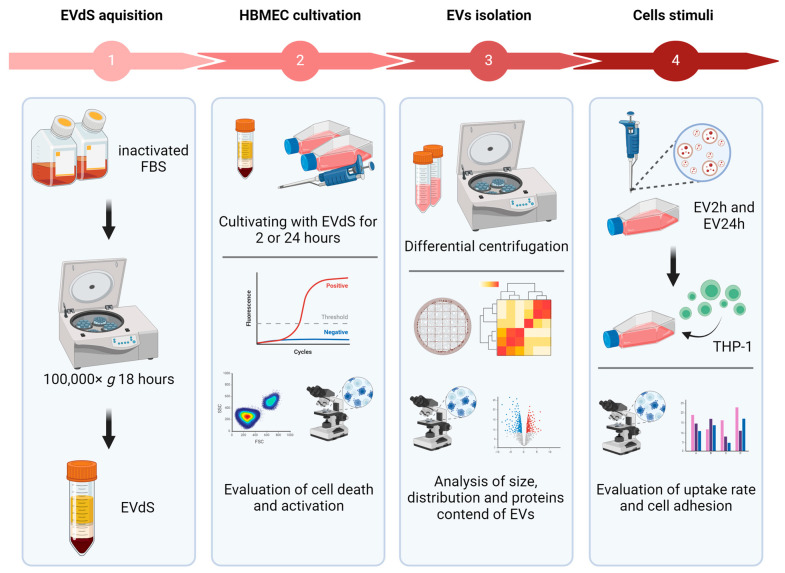
Experimental flowchart. Extracellular vesicle-depleted fetal bovine serum (EVdS) was obtained by centrifugation at 100,000× *g* (1). Afterward, the human brain microvascular endothelial cells (HBMECs) were cultured with EVdS for 2 or 24 h; cell death and activation were assessed by flow cytometry and quantitative PCR (qPCR) (2). EVs isolated from cells cultured for 2 h (EV2h) and EV24h were obtained by differential centrifugation, and their size, concentration, and protein content were evaluated by transmission electron microscopy (TEM), nanoparticle tracking analysis (NTA) and liquid chromatography–tandem mass spectrometry (LC–MS/MS) (3). HBMECs were incubated with EV2h or EV24h to evaluate the uptake rate, expression of adhesion molecules, and adhesion to human acute monocytic leukemia cell line (THP-1) cells (4). Figure created with https://help.biorender.com/en/ accessed on 6 April 2024.

The experimental flowchart summarizing the experiments described below is shown in Figure 8, while a table with all the abbreviations can be accessed from Supplementary Table S2.

### 4.1. Cell Culture

Human brain microvasculature endothelial cells (HBMECs) were maintained in 75 cm^2^ (T75) culture flasks with 12 mL of Dulbecco Modified Eagle Medium (DMEM) (Ref. 12100046 Gibco, Billings, MT, USA) supplemented with 10% FBS (Ref. 12657029, Gibco, USA), 2 mM L-glutamine, 50 IU/mL (Ref. 21051-024) penicillin and 50 μg/mL streptomycin (Ref. 15140122, Gibco, USA) at 37 °C and 5% CO_2_. Upon reaching 90% confluence, equivalent to approximately 1.4 × 10^6^ total cells, the cells were removed from the bottles with the addition of 2.5 mL of trypsin (5 mg/mL) (Ref. T4799-25 g, Sigma-Aldrich™, Saint Louis, MO, USA) + ethylenediaminetetraacetic acid (EDTA) (2 mg/mL) (Ref. E9884-100G, Sigma-Aldrich™, USA) and counted, after which approximately 7.5 × 10^6^ cells were transferred to new T75 flasks or used for experiments. THP-1 cells (ATCC TIB-202) were maintained in 25 cm^2^ (T25) culture flasks with 6 mL of Roswell Park Memorial Institute (RPMI) medium (Ref. 31800022 Gibco, USA) supplemented with 10% FBS, 50 IU of penicillin, 50 μg of streptomycin, and 2 mM L-glutamine at 37 °C and 5% CO_2_. After reaching a density of approximately 6.5 × 10^5^ total cells, the cells were centrifuged at 500× *g* for 5 min, after which approximately 1.0 × 10^5^ cells were transferred to a new T25 flask or used for experiments.

### 4.2. EV Isolation

For FBS-EV depletion, FBS was inactivated at 56 °C for 30 min, followed by centrifugation at 100,000× *g* for 18 h at 4 °C in an ultracentrifuge model CP80NX with rotor P40ST S/N 2666 (Eppendorf Himac Technologies, Ibaraki, Japan), sterilized by filtration through a 0.22 μm filter (Ref. SLGP033LS, Merck Millipore, St. Louis, MO, USA), and stored at −20 °C until use. For the isolation of EVs from HBMEC, cells were cultured in 150 cm^2^ (T150) flasks under the aforementioned conditions. After the cells reached approximately 90% confluence, they were washed with phosphate-buffered saline (PBS), and the culture medium was replaced with DMEM containing 10% FBS-depleted EVs (EVdS). Then, the cells were incubated for 2 or 24 h, and EVs were isolated by differential centrifugation at 300× *g* for 10 min, 2000× *g* for 10 min, 10,000× *g* for 30 min, and 100,000× *g* for 110 min twice. In the first three centrifugations, the supernatant was collected, and in the last centrifugation, the precipitate was suspended in 50 μL of sterile PBS and stored at 4 °C until use, for a maximum of 1 week. The supernatant from the last centrifugation was subjected to protein precipitation using ammonium sulfate (Ref. A4418; Sigma-Aldrich™, USA). Initially, ammonium sulfate was gradually added until the sample volume reached 40% saturation. The sample was subsequently centrifuged at 12,000× *g* for 10 min to collect the first fraction of the precipitate. Then, ammonium sulfate was added until 80% saturation was reached, and the sample was centrifuged again at 12,000× *g* for 10 min. The entire process was carried out at 4 °C.

### 4.3. Characterization of EVs

For nanoparticle tracking analysis (NTA), EVs obtained from HBMEC, as previously described, were diluted 1:10 in PBS to obtain an approximate final volume of 500 μL. The EV suspension was then collected using a sterile 1 mL syringe and injected into a NanoSight device (Malvern, London, UK) LM14 with a 532 nm laser module. Five technical replicates, each lasting 60 s, were performed.

To analyze EVs by transmission electron microscopy (TEM), samples of EVs suspended in approximately 50 μL of PBS were added to carbon-coated metallic grids (Ref. FF300-NI-50, EMS, Houston, TX, USA). Negative staining was performed with uranyl acetate at a final concentration of 5% (Ref. 22400, EMS, USA), and excess PBS or uranyl acetate was removed using a paper towel. Finally, the samples were visualized with a JEM-1400Plus transmission electron microscope (JEOL, Tokyo, Japan).

To evaluate the EVs by flow cytometry, the isolated particles were incubated with 4.5 μg/mL anti-CD63 (Cluster of Differentiation 63) antibody produced in rabbits (Ref. PA5-92370, Thermo Fisher Scientific, Waltham, MA, USA) and 1.7 μg/mL goat anti-rabbit IgG antibody (H + L) conjugated to Alexa Fluor^®^ 488 (Ref. A11008, Thermo Fisher Scientific, USA). The EVs were then washed to remove any residual antibodies through centrifugation at 100,000× *g* for 110 min. The antibody-labeled EVs were subsequently suspended in 500 μL of sterile PBS, resulting in a final concentration of approximately 1.0 × 10^6^ EV/mL. The evaluation of EVs was performed using CytoFLEX equipment from Beckman Coulter, Brea, CA, USA. The identification of EVs was conducted by reading the side violet laser scatter (V-SSC) in the reading channel (405/10). To ensure accuracy, Megamix-Plus SSC (Side Scatter) beads (Ref. 7803, BioCytex, Marseille, France) and Megamix-Plus FSC (Forward Scatter) beads (Ref. 7802, BioCytex, France) were used as references during the evaluation process. The gating strategy used in this study is described in Supplementary Figure S1.

For proteomic analysis, EVs were quantified by a Qubit protein assay (Ref. Q33211, Thermo Fisher, USA) and a fluorometer (Q33238, Thermo Fisher Scientific, USA) at wavelengths between 458 and 590 nm. Analysis by mass spectrometry was subsequently conducted with the application of 10 μg of total extract of EVs that were resolved in a 12% polyacrylamide gel. Electrophoresis was carried out over 120 min under a constant setting of 10 V/cm in the preparative gel and 15 V/cm in the resolving gel in a vertical Mini-Protean Tetra Cell tank (Ref. 1658004, Bio-Rad, Hercules, CA, USA) followed by silver nitrate staining (S5506, Sigma-Aldrich™, USA).

The regions of the gel containing the proteins were cut and dehydrated with absolute ethanol (Ref. 32221-1 L, Honeywell, Charlotte, NC, USA). After that, the samples were reduced with 10 mM dithiothreitol (DTT) (Ref. 19779, Sigma-Aldrich™, USA), digested with 12.5 ng/μL trypsin, and alkylated with 55 mM iodoacetamide (Ref. I6125, Sigma-Aldrich™, USA). The elution of peptides in Stage Tips was performed by adding 0.1% formic acid (Ref. 1002640100, Merck, Lebanon, NJ, USA) and centrifuging at 1000× *g* for 5 min. The analysis of the peptides was performed using an Eksigent nano LC 1d plus liquid chromatography instrument (Thermo Fisher Scientific, USA) coupled to an LTQ-Orbitrap XL ETD mass spectrometer (Thermo Fisher Scientific, USA). The identification of the spectra was performed using MaxQuant v2.5.1.0 software, and the data were analyzed using Perseus 2.0.11.0 (Max Planck Institute of Biochemistry, Planegg, Germany), R 4.2.2, and R-Studio 2023.06.1 +524 (Posit Software, Boston, MA, USA) software. The following packages were used for R-Studio: corrplot for correlation matrices; gplots and VennDiagram for Venn diagrams and heatmaps; factoextra for PCA; ggplot2 for volcano plots; and clusterProfiler, org.Hs.eg.db and AnnotationDbi for overrepresentation analysis (ORA). ORA was also evaluated by using the ShinyGO v.0.77 platform (Ge-Lab, Niskayuna, NY, USA) and was conducted based on the number of proteins, the enrichment of the pathways, and a false discovery ratio (FDR) cut-off of 0.05.

### 4.4. Evaluation of Endothelial Cell Activation and Death

T25 culture flasks were seeded with 2.5 × 10^5^ HBMEC under the aforementioned conditions. When the cells reached approximately 90% confluence, they were washed with sterile PBS and incubated for 2 or 24 h in DMEM supplemented with FBS or EVdS to evaluate the transcriptional profile, ICAM-1 expression, and cell death. For transcription evaluation, total RNA was extracted from cells with a RNeasy Micro Kit (Ref. 74004; Qiagen, Hilden, Germany), and cDNA was synthesized using a SuperScript™ II Reverse Transcriptase Kit (Ref. 18064022; Thermo Fisher Scientific, USA) following the manufacturer’s instructions. Specific oligonucleotides for *IL6* (F: 5′-ACTCACCTCTTCAGAACGAATTG-3′ and R: 5′-CCATCTTTGGAAGGTTCAGGTTG-3′), *IL8* (F: 5′-TCTGCAGCTCTGTGTGAAGG-3′ and R: 5′-ACTTCT CCACAACCCTCTGC-3′), *suppressor of cytokine signaling 3 (SOCS3*) (F: 5′-GGCCACTCTTCAGCATCTC-3′ and R: 5′-ATCGTACTGGTCCAGGAACTC-3′), *vascular endothelial growth factor (VEGF*) (F: 5′-TGCAGATTATGCGGATCAAACC-3′ and R: 5′-TGCATTCACATTTGTTGTGCTGTAG-3′), and *OCCLUDIN* (F: 5′-AAGACGATGAGGTGCAGAAG-3′ and 5′-GTGAAGAGAGCCTGACCAAA-3′) were used for evaluation by quantitative PCR (qPCR). Specific oligonucleotides for *glyceraldehyde-3-phosphate dehydrogenase* (*GAPDH*) (5′-GGCCTCCAAGGAGTAAGACC-3′ and R: 5′-GACTGAGTGTGGCAGGGACT-3′) were used for normalization. SYBR™ Select Master Mix (Ref. 4472908, Applied Biosystems™, San Francisco, CA, USA) was used according to the manufacturer’s recommendations.

For the evaluation of intercellular adhesion molecule 1 (ICAM-1) expression, HBMECs were cultured in DMEM supplemented with or without 10% FBS or EVdS for 2 or 24 h. After incubation, the cells were fixed with 4% paraformaldehyde (Ref. 694, Vetec, Sao Paulo, Brazil), incubated with 5 μg/mL anti-ICAM-1 antibody produced in mice (Ref. MA1-19028, Thermo Fisher Scientific, USA), incubated with 1.7 μg/mL secondary goat anti-mouse IgG (H + L) conjugated to Alexa Fluor^®^ 488 (Ref. A11001, Thermo Fisher Scientific, USA), and evaluated under a DMi80 fluorescence microscope (Leica Microsystem, Wetzlar, Germany). To evaluate the integrated density, the images obtained by microscopy were converted to an 8-bit format, and the pixels were quantified using ImageJ 1.53k software (National Institutes of Health, Osaka, Japan).

For the assessment of cell death, approximately 1.0 × 10^5^ cells were transferred to a 96-well plate, stained with Alexa Fluor^®^ 488 Annexin V (1:100) and 1.0 μg/mL propidium iodide (PI) (Ref. V13245; Thermo Fisher Scientific, USA), and evaluated by flow cytometry using a FACSCanto II (Becton Dickinson, Franklin Lakes, NJ, USA). As a positive control for this analysis, we used HBMEC incubated with 10 ng/mL of tumor necrosis factor-alpha (TNF-α).

### 4.5. Evaluation of the Biological Activity of EVs

To assess EV uptake, HBMEC were incubated with CellTrace™ carboxyfluorescein succinimidyl ester (CFSE) (Ref. C34554; Thermo Fisher Scientific, USA) at a final concentration of 5 μM for 20 min in the dark. Subsequently, the excess CFSE was removed by washing with sterile PBS, and the EV isolation process was conducted following the previously mentioned method. HBMEC were cultivated in 24-well plates and allowed to reach approximately 90% confluence. The cells were then treated with 100 ng/mL EV2h or EV24h for 30 min, 1 h, or 4 h. Following the incubation period, the cells were fixed with paraformaldehyde (4%) and stained with 1.0 μg/mL 4′,6-diamidino-2-phenylindole (DAPI) (Ref. D1306, Thermo Fisher Scientific, USA) at a final concentration of 10 μM for 15 min. The cells were evaluated using a DMi80 fluorescence microscope (Leica Microsystems, Germany). The acquired images were analyzed using ImageJ 1.53k software.

To assess cell adhesion, we used human acute monocytic leukemia cell line (THP-1) cells, which are well studied for their ability to adhere to endothelial cells through adhesion molecules [73,121]. For this experiment, it was necessary to culture HBMEC in RPMI instead of DMEM to avoid any changes in the THP-1 cells that must be cultured in RPMI. HBMEC and THP-1 cells were maintained in RPMI medium supplemented with 10% FBS, 50 U of penicillin, 50 μg of streptomycin, and 2 mM L-glutamine. Approximately 4.0 × 10^4^ HBMEC were grown in 24-well plates and incubated with 100 ng/mL of EV2h or EV24h, 10 ng/mL of TNF-α (Ref. A42552, Invitrogen, Carlsbad, CA, USA), or RPMI for 24 h. One hour before the adhesion assay, another group of HBMEC was incubated for 1 h with 1 μg/mL primary anti-ICAM-1 antibody and washed to remove unbound antibodies. After that, the THP-1 cells were incubated with 5 µM CellTrace™ CFSE, washed with sterile PBS, and cocultured with HBMEC at a 1:1 ratio for 1 h. At the end of the incubation step, the cells were washed at least three times to remove nonadherent THP-1 cells, fixed with 4% paraformaldehyde, labeled with DAPI, and evaluated under a DMi80 fluorescence microscope (Leica, Germany).

### 4.6. Statistical Evaluation

Statistical tests were conducted based on the characteristics of each sample group. The Shapiro–Wilk test was used to assess sample normality at a significance level of 0.05. Differences in variance between samples were evaluated using Levene’s test at a significance level of 0.05. Pairwise comparisons of samples with a normal Gaussian distribution (parametric) were performed using either simple or multiple unpaired *t*-tests. For multiple unpaired analyses with a normal distribution and equal variance, analysis of variance (ANOVA) was conducted, followed by Tukey’s test; when the data were not normally distributed, the Kruskal–Wallis test was used, followed by Dunn’s test.

A breakdown of the specific statistical tests employed can be found in the respective figure legends. All the statistical analyses were carried out using GraphPad Prism 8.0 Software (GraphPad Software, Inc., San Diego, CA, USA) and R 4.2.2. Statistical significance was determined for analyses with a *p*-value or probability of significance equal to or less than 0.05.

## 5. Conclusions

In conclusion, HBMECs cultivated with EVdS produce EVs with different physical characteristics and protein contents depending on the cultivation interval. Although our studies have provided important insights into cellular communication mediated by EVs, the complexity of the information transmitted by these particles demands further research for a complete understanding. Through our studies, we aim to contribute to the establishment of a well-founded understanding of EV-mediated cellular communication.

## Data Availability

All data generated or analyzed during this study are included in this published article and its Supplementary Materials.

## Supplementary Materials

The following supporting information can be downloaded at: https://www.mdpi.com/article/10.3390/ijms25094761/s1.
