# Supplementary material for: Unraveling the Impact of Extracellular Vesicle-Depleted Serum on Endothelial Cell Characteristics over Time
Source: Int J Mol Sci. 2024 Apr 27;25(9):4761. doi: 10.3390/ijms25094761 (PMC11084606; doi:10.3390/ijms25094761)
Supplement: Supplementary file 1 [file ijms-25-04761-s001.zip › ijms-2907457-supplementary.pdf]

Title: *Unraveling the impact of extracellular vesicle-depleted serum on endothelial cell characteristics over time*

Authors: Luiz Fernando Cardoso Garcia[1]; Pryscilla Fanini Wowk[2], Letusa Albrecht[1,*]

Affiliation: 1: Laboratório de Pesquisa em Apicomplexa, ICC-Fiocruz-PR. 2: Grupo de Pesquisa em Imunologia Celular, ICC-Fiocruz-PR.

***:** corresponding author.

SUPPLEMENTARY DATA AND SUBTITLES

Unique proteins in Sup2h	Common between EV2h and Sup2h	Unique proteins in Sup24h	Common between EV24h and Sup24h
GRHPR	RPL4	TAF15	FASN
TXNRD1	RPL3	SET	CCT5
NACA	RPS8	SPTAN1	IPO7
RPS5	RPS3A	PROS1	S100A6
LAP3	CLTC	HIST1H2AC	THBS1
RPS25	EEF1A1	ASAH1	XRCC6
	HSPA1B	DSC2	AKR1B1
	ENO1	CTSV	TNC
	EEF1A2	NUDT5	TARS
	GPI	NPC2	PRDX6
	LDHB	NRP1	ATIC
	HSPD1	PTPRK	ACLY
	ACTN1	B4GAT1	TINAGL1
	P4HB	ATRN	CLTC
	VCP	PRSS23	EEF1A1
	HSP90B1	HPRT1	HSPA1B
	SLC3A2	CBS	ENO1
	PPIB	TXN	EEF1A2
	FLNB	TIMP2	GPI
	PDIA3	CBR1	LDHB
	MYH9	GLB1	HSPD1
	ACTN4	PAM	ACTN1
	UBC	WARS	P4HB
	HNRNPC	SFN	VCP
	RAN	GPC1	HSP90B1
	MYL6	BTD	SLC3A2
	RPLP0	LAMB2	PPIB
	ARPC4-TTLL3	NEO1	FLNB

CD44	GDF15	PDIA3
EIF5A	ROBO1	MYH9
ALDOA	RNASET2	ACTN4
CLIC1	CTSH	UBC
ADAM10	LFNG	HNRNPC
LDHA	LIPA	RAN
PGK1	ARSA	MYL6
FN1	GBA	RPLP0
GAPDH	CLN5	ARPC4-TTLL3
ANXA2	PPT1	CD44
TUBB	MANBA	EIF5A
PFN1	C1R	ALDOA
HSP90AA1	GLA	CLIC1
HSP90AB1	SDF4	ADAM10
VIM	CTSF	LDHA
LGALS1	ATP6AP1	PGK1
HSPA5	CTSL	FN1
HSPA8	AGT	GAPDH
EEF2	TCN2	ANXA2
PKM	HAVCR1	TUBB
EZR	MAN2B2	PFN1
NME1	CTSB	HSP90AA1
TCP1	NUCB2	HSP90AB1
VCL	FTH1	VIM
PGAM1	PLA2G15	LGALS1
FLNA	TWSG1	HSPA5
UBA1	DNASE2	HSPA8
AHCY	LAMA5	EEF2
MSN	FSTL3	PKM
TKT	FUCA1	EZR
YWHAB	GUSB	NME1
COL18A1	GAA	TCP1
MDH2	COL6A2	VCL
IQGAP1	BMP1	PGAM1
GDI2	NID1	FLNA
CCT8	NAGA	UBA1
CCT4	GM2A	AHCY
TPI1	EFNA1	MSN
ACTB	PTPRG	TKT
EIF4A1	HSPA13	YWHAB
HNRNPK	SGSH	COL18A1
YWHAE	SIRPA	MDH2
RPS4X	JAG1	IQGAP1
HIST1H4A	SRPX	GDI2
PPIA	CTBS	CCT8
YWHAZ	GALNT2	CCT4

ACTA1	PTPRS	TPI1
TUBA1B	GAS6	ACTB
TUBB4B	WFDC2	EIF4A1
CCT2	MBTPS1	HNRNPK
YWHAH	EXT1	YWHAE
PRDX1	MAN2A1	RPS4X
DHX9	QPCT	HIST1H4A
KPNB1	MEGF8	PPIA
FSCN1	GALNT7	YWHAZ
PADI2	CANT1	ACTA1
TLN1	PVRL2	TUBA1B
CTSD	PTPRU	TUBB4B
LMNA	EXT2	CCT2
NPEPPS	NEU1	YWHAH
NCL	SORT1	PRDX1
RPS2	OSMR	DHX9
RPS16	CHID1	KPNB1
GOT2	XYLT2	FSCN1
PDIA4	SIAE	PADI2
RPS3	DPP7	TLN1
EEF1G	MINPP1	CD9
CCT6A	PLS3	ITGB1
B2M	SNRPN	HIST1H2BN
YWHAG	PEBP1	ICAM1
CRYZ	PGM1	NPNT
GANAB	HDGF	SDCBP
	KARS	SERPINE2
	PLEC	S100A11
	UGP2	SEMA3C
	VEGFA	
	SGCE	
	PCOLCE2	
	TGFB1	
	FAM49B	
	EIF4A2	
	MAN1B1	
	TOR1B	
	B4GALT5	
	IDS	
	EFNB3	
	PLD3	
	HTRA1	
	CCT7	
	GNPTG	

Supplementary table S1. Proteins unique to Sup2h and Sup24h and in common with EV2h and EV24h. EVs were obtained by ultracentrifugation from HBMEC grown in

DMEM supplemented with EVdS for 2 or 24 hours. After isolation of EVs, supernatant proteins were precipitated with ammonium sulfate and both EV and supernatant proteins were resolved on a polyacrylamide gel and analyzed using LC-MS/MS. The reference proteome for *Homo sapiens* was acquired from the UniProt database. Distribution of unique or common proteins between EVs and supernatant depleted from EVs at 2h (Sup2h) or 24h (Sup24h) was expressed in a spreadsheet, with 7 unique proteins found in Sup2h, 101 common between Sup2h and EV2h, 119 unique proteins found in Sup24h and 104 common between Sup24h and EV24h. The data was obtained from three biological replicates.

**Abbreviation**	**Definition**
EV	Extracellular Vesicle
Ab-ICAM-1	Antibody anti ICAM-1
ACTG1	Actin Gamma-1
ANOVA	Analisys of Aariance
ANX5	Annexin-V
ANXA1	Annexin A1
ANXA5	Annexin A5
ARF6	ADP-ribosylation factor 6
ATP5B	ATP synthase F1 subunit beta
BCL2	B-cell lymphoma 2
CD63	Cluster of Diferentiation 63
CHO	Chinese Hamster Ovary
CLTC	Clathrin heavy chain 1
COL18A1	Collagen Type XVIII Alpha 1 Chain
DAPI	4',6-diamidino-2-phenylindole
DARC	Duffy antigen receptor for chemokine
DTT	Dithiothreitol
EDTA	ethylenediaminetetraacetic acid
ER	Endoplasmatic Reticulum
ERM	Ezrin, Radixin and Moesin
ESYT1	extended synaptotagmin-1
EV24h	Extracellular vesicles isolated from a 24-hour cultivation
EV2h	Extracellular vesicles isolated from a 2-hour cultivation
EVdS	Extracellular Vesicle depleted Serum
FBS	Fetal Bovine Serum
FDR	Foward Scatter
FEA	Functional Enrichment Analysis
FN1	Fibronectin-1
HBMEC	Human Brain Microvascular Endothelial Cell
HIV-1	Human Immunodeficiency Virus 1
HSP70	Heatshock Protein 70
HSPD1	Heat Shock Protein Family D Member 1
ICAM-1	Intercellular Adhesion Molecule-1
IL-6	Interleukin-6

IL-8	Interleukin-8
ITGB1	Integrin Beta 1
KEGG	Kyoto Encyclopedia of Genes and Genomes
LC-MS/MS	Liquid Chromatography Tandem Mass Spectrometry
lEV	large Extracellular Vesicle
LFQ	Label-Free Quantification
MFGE8	Milk fat globule-EGF factor 8
MFI	Mean Fluorescence Intensity
MISEV	Minimal Information for Studies of Extracellular Vesicles
mTORC1	mechanistic target of rapamycin Complex 1
NF-κB	Nuclear Factor Kappa B
NTA	Nanopartycle Tracking Analisys
ORA	overrepresentation analysis
P4HB	prolyl 4-hydroxylase
PCA	Principal Component Analysis
PDCD6IP	Programmed cell death 6-interacting protein
PDIA3	Protein disulfide isomerase A3
PI	propidium iodide
PI3K	Phosphoinositide 3-kinase
qPCR	quantitative PCR
RAP1	Ras-related protein 1
RPL4	Ribosomal Protein L4
RPL6	Ribosomal Protein L6
RPMI	Roswell Park Memorial Institute
SDCBP	Syndecan Binding Protein
sEV	small Extracellular Vesicle
SOCS3	Suppressor of Cytokine Signaling 3
SSC	Side Scatter
Sup24h	Supernatant from a 24-hour cultivation
Sup2h	Supernatant from a 2-hour cultivation
T150	150 cm2
T25	25 cm2
T75	75 cm2
TEM	Transmission Electron Microscopy
TGF-B	Tissue Growth Factor B
TGM2	Transglutaminase 2
THP-1	human acute monocytic leukemia cell line-1
THP-1	Human Acute Monocytic Leukemia Cell Line
TNF	Tumor Necrosis Factor
TNFr	TNF recepctor
TUBA1	Tubulin A1
TUFM	Mitochondrial Tu translation elongation factor
VEGF	Vascular Endothelial Growth Factor
V-SSC	Violet Side Scatter

Supplementary table S2. List of abbreviations.

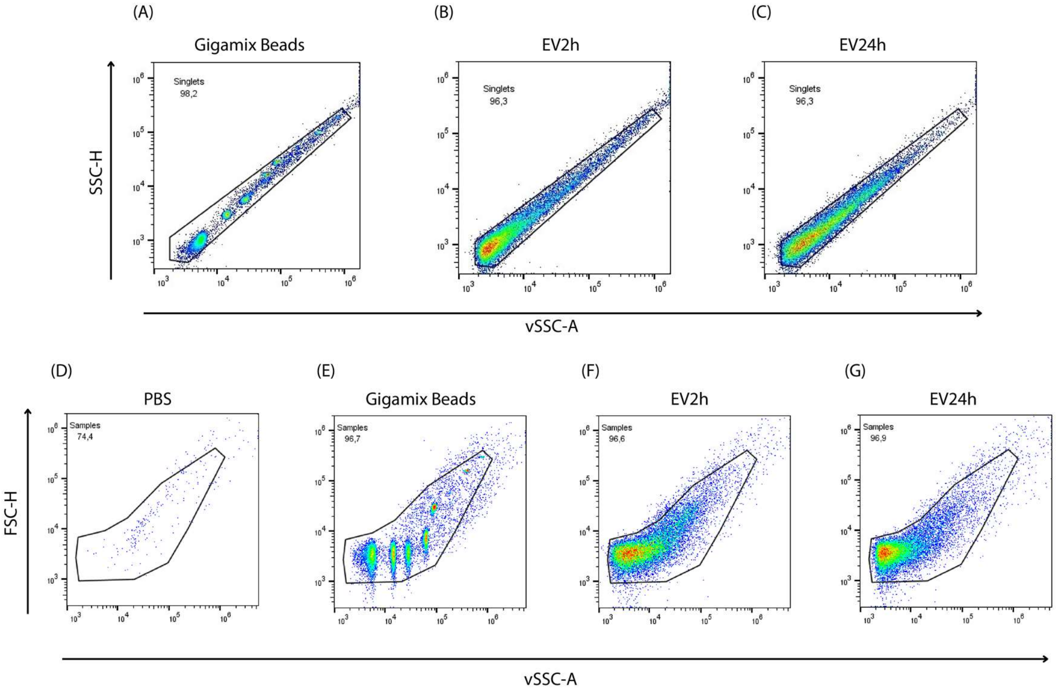

Supplementary figure S1. Gating strategy for identifying EVs by flow cytometry. EVs were obtained by differential centrifugation of the supernatant of HBMECs grown in DMEM with EVdS for 2 and 24 hours. After that, they were evaluated by flow cytometry. Initially, Gigamix Bead (Megamix-Plus SSC beads and Megamix-Plus FSC beads) (**A**), EV2h (**B**) and EV24h (**C**) singlets were identified and selected by the relationship between height (SSC-H) and area (vSSC-A) measured from the lateral light scattering. The second gate was developed taking as reference the relationship between forward (FSC-H) and side (vSSC-A) light scattering of the Gigamix Bead (**D-G**). Graphs constructed from a representative replicate of three biological replicates.

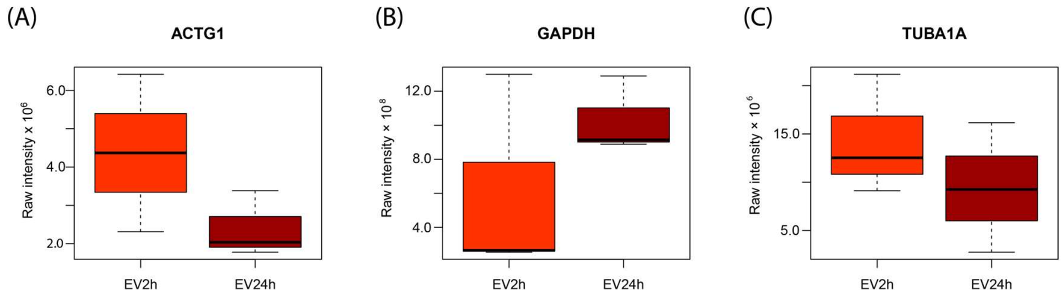

Supplementary figure S2. Evaluation of proteins with constitutive expression in EV2h and EV24h. Extracellular vesicles obtained by ultracentrifugation from HBMEC grown in DMEM supplemented with EVdS for 2 or 24 hours. Total EV protein extract resolved on polyacrylamide gel and analyzed on LC-MS/MS. Reference proteome for *Hommo sapiens* acquired from the UniProt database. ACTG1 (**A**), GAPDH (**B**) and TUBA1A (**C**) were graphed based on their raw intensity. The data was obtained from three biological replicates.

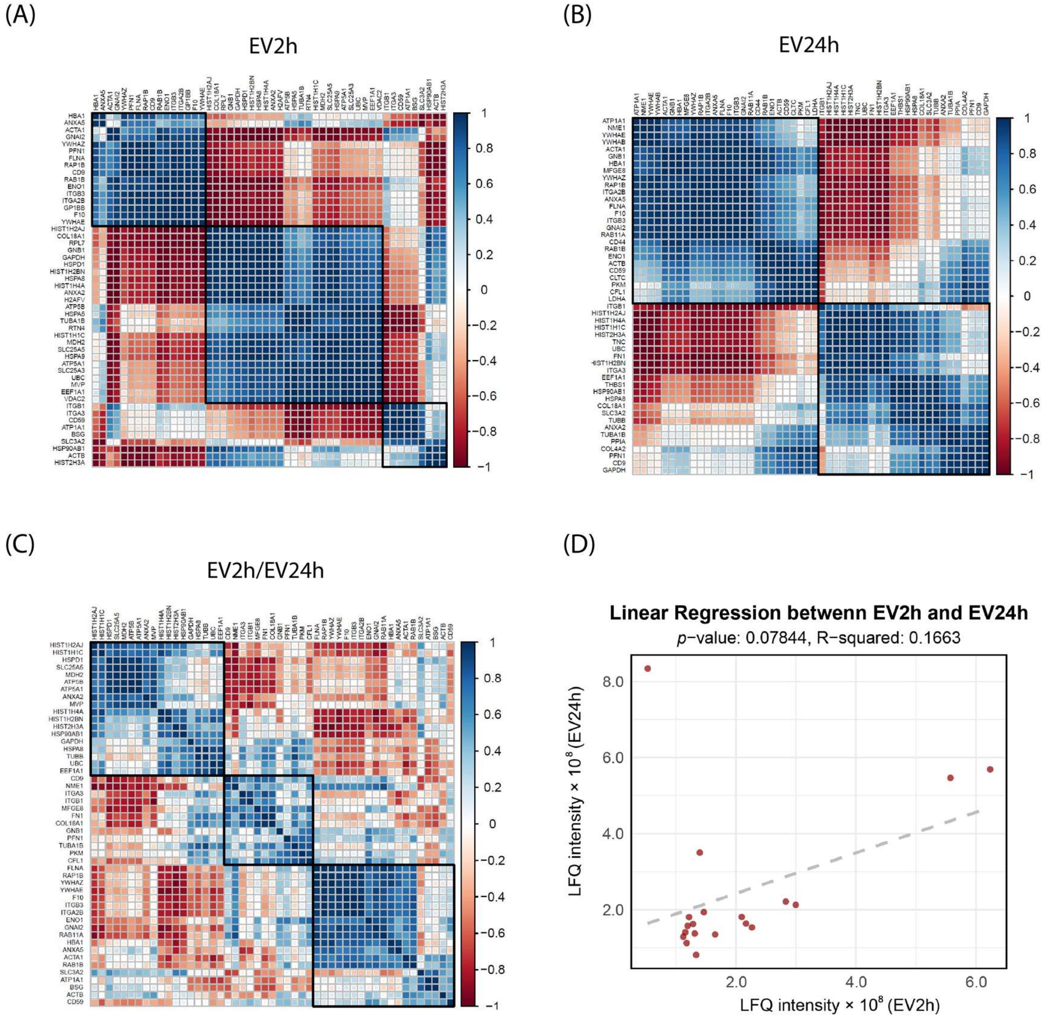

Supplementary figure S3. EV2h and EV24h exhibit distinct protein content. Extracellular vesicles were obtained by ultracentrifugation from HBMEC cells cultured in DMEM supplemented with EVdS for 2 or 24 hours. The total protein extract from EVs was resolved on a polyacrylamide gel and analyzed using LC-MS/MS. The reference proteome for *Homo sapiens* was acquired from the UniProt database. Top 50 proteins ranked based on LFQ intensity from EV2h (**A**), EV24h (**B**), and common proteins between EV2h and EV24h (**C**) were represented in correlation matrices. Graphs were organized using hierarchical clustering. 50 common proteins ranked according to LFQ intensity were plotted on a linear regression curve (**D**). Data were obtained from three biological replicates. 95% confidence interval.

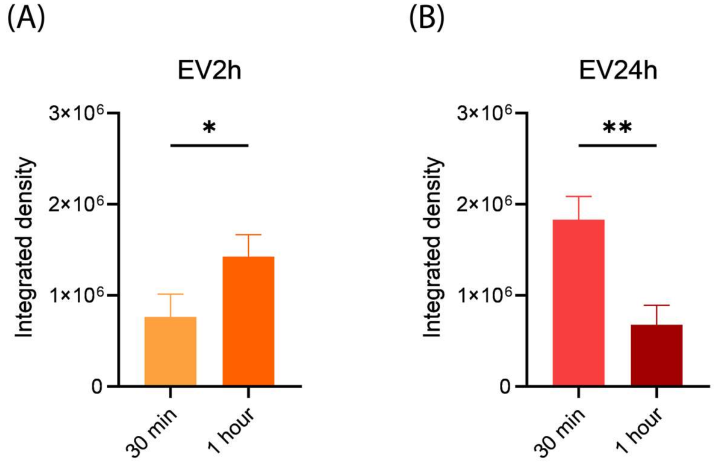

Supplementary Figure S4. EV2h and EV24h Uptake rate at 30 minutes and 1 hour. EVs were obtained from HBMEC previously labeled with CFSE and cultured in DMEM supplemented with EVdS. The isolation of EVs was performed by differential centrifugation. HBMEC were cultured in 24-well plates and incubated with EV2h (**A**) or EV24h (**B**) at a concentration of 100 ng/mL for 30 minutes or 1 hour. Subsequently, the cells were washed, fixed with 4% paraformaldehyde, stained with DAPI, and examined under a fluorescence microscope. The acquired images were analyzed using specific software, and the results were expressed as integrated density. The data were obtained from three biological replicates with five technical replicates each and are presented as mean ± standard deviation (t-test). *: p-value < 0.05; **: *p*-value < 0.01.

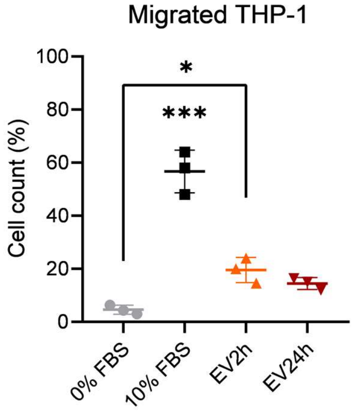

Supplementary Figure S5. EV2h exhibits chemotactic properties for THP1 cells. EVs were obtained from HBMEC cultured in RPMI supplemented with EVdS. Isolation of EVs was performed by differential centrifugation. Approximately 4.4×10^4 THP-1 cells were maintained in RPMI without FBS in the upper chamber of transwell plates with 8 µm polycarbonate filters. RPMI medium without FBS (0% FBS) or supplemented with 10% FBS, EV2h, or EV24h (100 ng/mL) was added to the lower chamber. After 16 hours of incubation, cells in the lower chamber were counted. Data were obtained from three biological replicates, three technical replicates, and expressed as mean ± standard deviation (ANOVA/Tukey); *: *p*-value < 0.05; ***: *p*-value < 0.001.

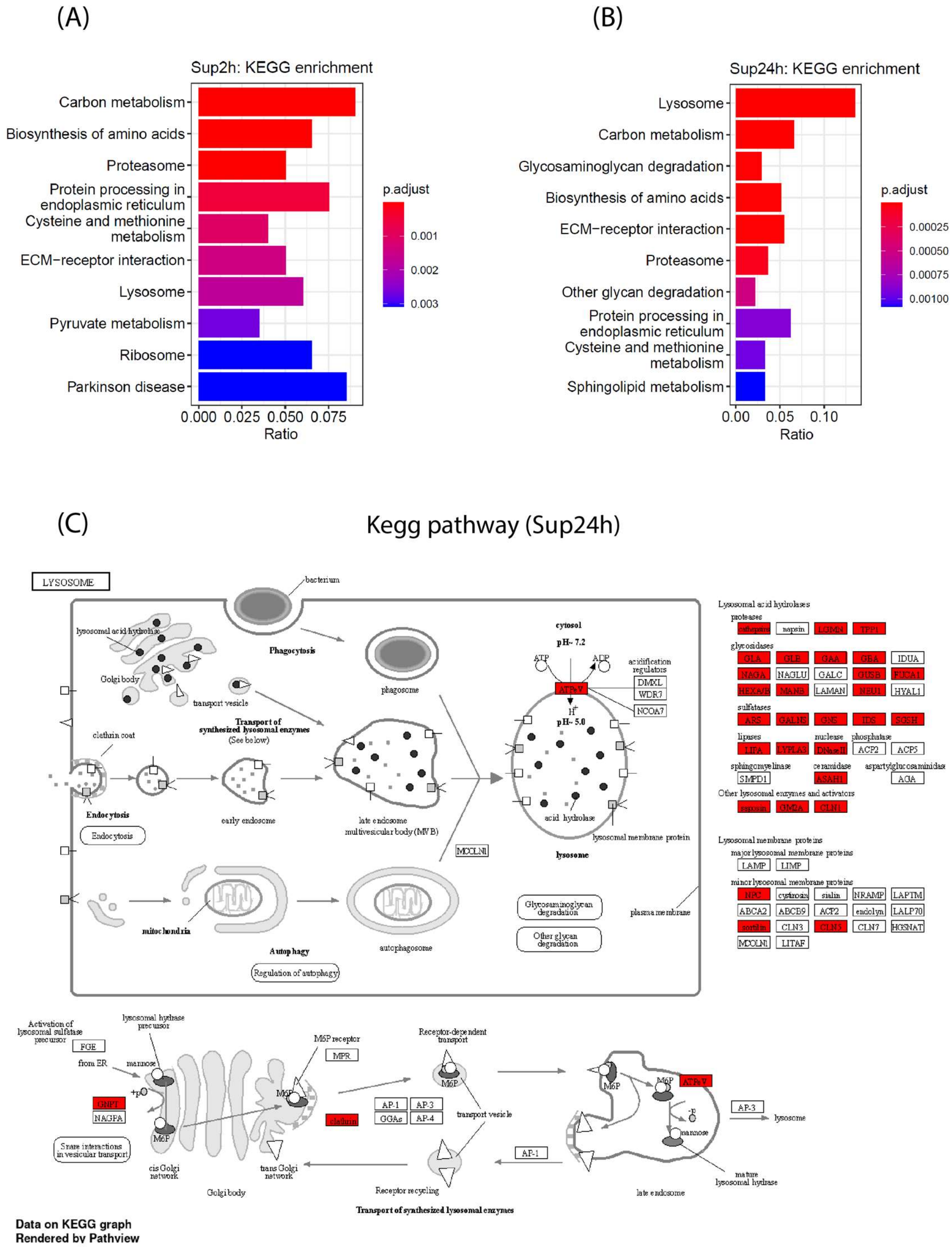

Supplementary figure S6. Pathway mapping of Sup2h and Sup24h. After isolation of HBMEC-EVs, supernatant proteins were precipitated with ammonium sulfate, resolved on polyacrylamide gel and analyzed by LC-MS/MS. Reference proteome for *Hommo sapiens* acquired from the UniProt database. KEGG terms of all proteins identified in Sup2h (**A**) or Sup24h (**B**) were functionally grouped by kappa score and shown in bar graphs. Enrichment analysis of genetic ontology and molecular interactions network was performed by ShinyGo and Pathview platforms (**C**). Red squares represent proteins found

in Sup24h. The significance of each term was demonstrated as $-\log_{10}$(FDR). Data obtained from three biological replicates.

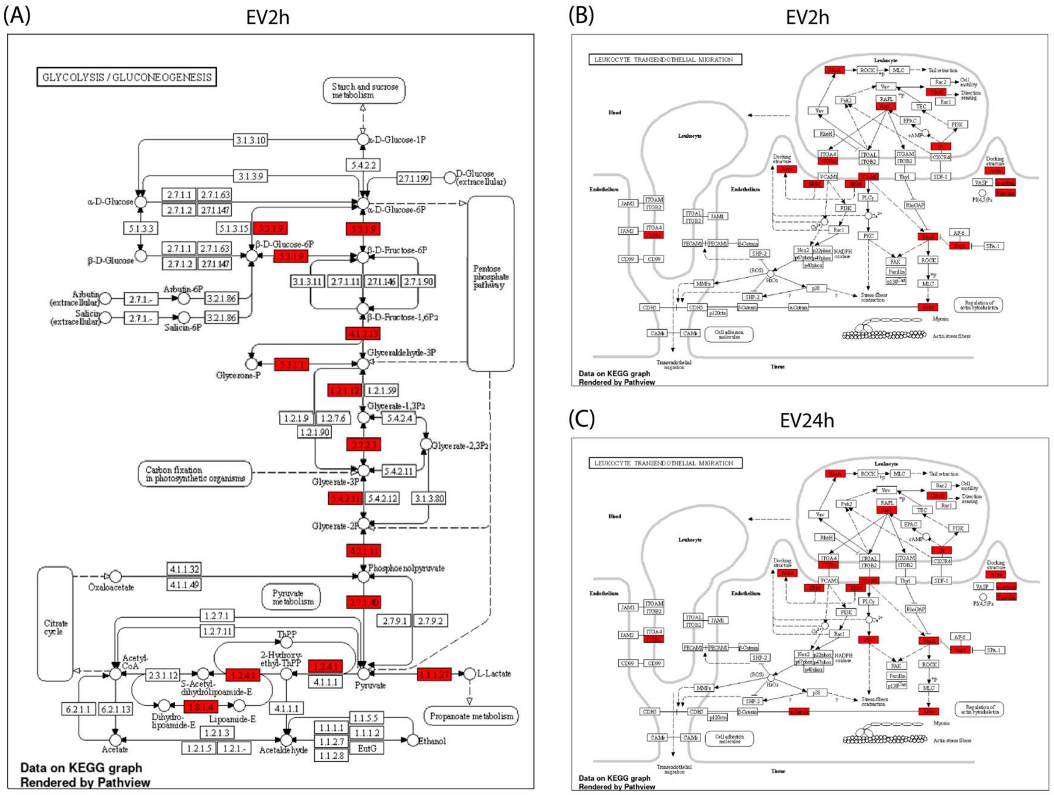

Supplementary figure S7. Molecular interaction network in the energy metabolism of EV2h proteins. EVs were obtained from HBMEC cultured in DMEM supplemented with EVdS for 2 or 24 hours using ultracentrifugation. Total protein extracts from the EVs were separated on a polyacrylamide gel and subsequently analyzed using LC-MS/MS. A reference proteome for *Homo sapiens* was acquired from the UniProt database. Molecular interaction networks for glycolysis/gluconeogenesis (**A**) and leukocyte transendothelial migration (**B** and **C**) were conducted using the ShinyGo and Pathview platforms. Proteins found in EV2h (**A** and **B**) and EV24h (**C**) are represented by red squares in the molecular interaction network. Data obtained from three biological replicates.

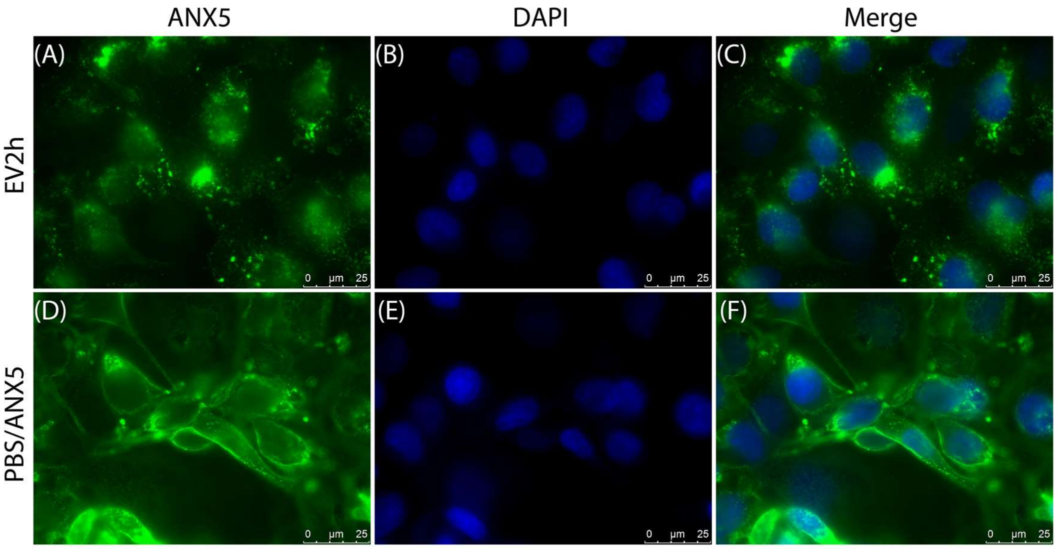

Supplementary figure S8. Evaluation of the uptake rate of EV2h labeled with ANX5. EVs were obtained from HBMEC cultured for 2 hours in DMEM supplemented with EVdS. The isolation of EV2h was performed by differential centrifugation. Subsequently, EV2h was labeled with Annexin-V conjugated to phycoerythrin (green), washed with sterile PBS, and centrifuged at 100,000× g (**A-C**). As a negative control, EV free Annexin-V stained PBS (PBS/ANX5) (**D-F**) was used. HBMEC were grown in 24-well plates and incubated with EV2h or PBS/ANX5 for 30 minutes. After, the cells were washed, fixed with 4% paraformaldehyde, stained with DAPI (blue), and then evaluated under a fluorescence microscope. Representative images of three biological replicates.
